# Embigin Promotes Prostate Cancer Progression by S100A4-Dependent and-Independent Mechanisms

**DOI:** 10.3390/cancers10070239

**Published:** 2018-07-23

**Authors:** I Made Winarsa Ruma, Rie Kinoshita, Nahoko Tomonobu, Yusuke Inoue, Eisaku Kondo, Akira Yamauchi, Hiroki Sato, I Wayan Sumardika, Youyi Chen, Ken-Ichi Yamamoto, Hitoshi Murata, Shinichi Toyooka, Masahiro Nishibori, Masakiyo Sakaguchi

**Affiliations:** 1 Department of Cell Biology, Okayama University Graduate School of Medicine, Dentistry and Pharmaceutical Sciences, Okayama 700-8558, Japan; ruma_winarsa@yahoo.co.id (I.M.W.R.); rie-k@okayama-u.ac.jp (R.K.); pl5i0zd8@s.okayama-u.ac.jp (N.T.); sdikarox@yahoo.co.uk (I.W.S.); p4qp2jhb@s.okayama-u.ac.jp (Y.C.); pzhc98l5@okayama-u.ac.jp (K.-I.Y.); murata@md.okayama-u.ac.jp (H.M.); 2 Department of Biochemistry, Faculty of Medicine, Udayana University, Denpasar 80232, Bali, Indonesia; 3 Faculty of Science and Technology, Division of Molecular Science, Gunma University, Gunma 376-8515, Japan; yinoue@gunma-u.ac.jp; 4 Division of Molecular and Cellular Pathology, Niigata University Graduate School of Medicine and Dental Sciences, Niigata 951-8510, Japan; ekondo@med.niigata-u.ac.jp; 5 Department of Biochemistry, Kawasaki Medical School, Okayama 701-0192, Japan; akiray@med.kawasaki-m.ac.jp; 6 Departments of Thoracic, Breast and Endocrinological Surgery, Okayama University Graduate School of Medicine, Dentistry and Pharmaceutical Sciences, Okayama 700-8558, Japan; maddux31glavine47@yahoo.co.jp (H.S.); toyooka@md.okayama-u.ac.jp (S.T.); 7 Department of Pharmacology, Faculty of Medicine, Udayana University, Denpasar 80232, Bali, Indonesia; 8 Department of Pharmacology, Okayama University Graduate School of Medicine, Dentistry and Pharmaceutical Sciences, Okayama 700-8558, Japan; mbori@md.okayama-u.ac.jp

**Keywords:** extracellular S100A4, embigin, AMPK, mTORC1, prostate cancer progression

## Abstract

Embigin, a transmembrane glycoprotein belonging to the immunoglobulin superfamily, is involved in prostate and mammary gland development. As embigin’s roles in cancer remain elusive, we studied its biological functions and interaction with extracellular S100A4 in prostate cancer progression. We found by a pull-down assay that embigin is a novel receptor for S100A4, which is one of the vital cancer microenvironment milleu. Binding of extracellular S100A4 to embigin mediates prostate cancer progression by inhibition of AMPK activity, activation of NF-κB, MMP9 and mTORC1 signaling, and inhibition of autophagy, which increase prostate cancer cell motility. We also found that embigin promotes prostate cancer growth, spheroid- and colony-forming ability, and survival upon chemotherapy independently of S100A4. An in vivo growth mouse model confirmed the importance of embigin and its cytoplasmic tail in mediating prostate tumor growth. Moreover, embigin and p21^WAF1^ can be used to predict survival of prostate cancer patients. Our results demonstrated for the first time that the S100A4-embigin/AMPK/mTORC1/p21^WAF1^ and NF-κB/MMP9 axis is a vital oncogenic molecular cascade for prostate cancer progression. We proposed that embigin and p21^WAF1^ could be used as prognostic biomarkers and a strategy to inhibit S100A4-embigin binding could be a therapeutic approach for prostate cancer patients.

## 1. Introduction

Embigin has been widely known as developmentally expressed protein that is involved in prostate and mammary gland development [1] and in the early stages of mouse embryogenesis [2]. Its expression has been found within the hematopoietic system including primitive hematopoietic cells [3] and as one of niche factors that regulate the localization and quiescence of hematopoietic stem/progenitor cells [4]. However, the biological functions of embigin in cancer remain elusive. Embigin expression has been reported to suppress tumorigenesis in breast cancer cells [5], while it promotes pancreatic cancer progression [6]. As well as its obscure role in cancer, a ligand for embigin has not been identified. In this study, we tried to identify a novel ligand of embigin and to determine the importance of embigin in cancer progression.

Cancer progression is associated to extracellular S100A4’s autocrine and paracrine functions [7]. Notably, S100A4 secretion by fibroblasts has been shown to be stimulated by co-culture with tumor cells in vitro [8], and IL-1β-expressing cancer cells showed a dramatic increase of S100A4 in the stroma adjacent to skeletal metastases, that promoting early colonization of prostate cancer in vivo [9]. These findings indicate that extracellular S100A4 functions as one of the vital cancer microenvironment milieu in cancer progression with intricate interplay between cancer and stromal cells for its secretion.

Extracellular S100A4 binds with various cell surface molecules including receptor for advanced glycation end products (RAGE), annexin II, and heparan sulfate proteoglycans [10,11,12]. Several studies reported S100A4-induced cell motility [13], capillary-like growth [8], and neurite outgrowth occur by a RAGE-independent mechanism [11]. These suggest the presence of an additional unknown receptor(s) that has a critical role in S100A4 biological functions.

In this study, we investigated the importance of embigin and S100A4 in cancer progression. Herein, we report for the first time that embigin acts as a novel receptor for extracellular S100A4. The binding of extracellular S100A4 to embigin mediates prostate cancer progression by orchestrating multiple mechanisms including inhibition of AMPK activity, activation of NF-κB, MMP9, and mTORC1 signaling, and inhibition of autophagy, which lead to an increase in cellular motility. Embigin also promotes prostate cancer growth, spheroid-and colony-forming ability, and survival upon chemotherapy independently of S100A4. Therefore, targeting S100A4/embigin is a promising therapeutic strategy for prostate cancer. Finally, survival analysis using publicly available data revealed that embigin and p21^WAF1^ provide potential benefit as prognostic biomarkers for prostate cancer patients.

## 2. Results

### 2.1. Embigin Expression Level Is High in Prostate Cancer and Embigin Binds to S100A4

**Figure 1:**
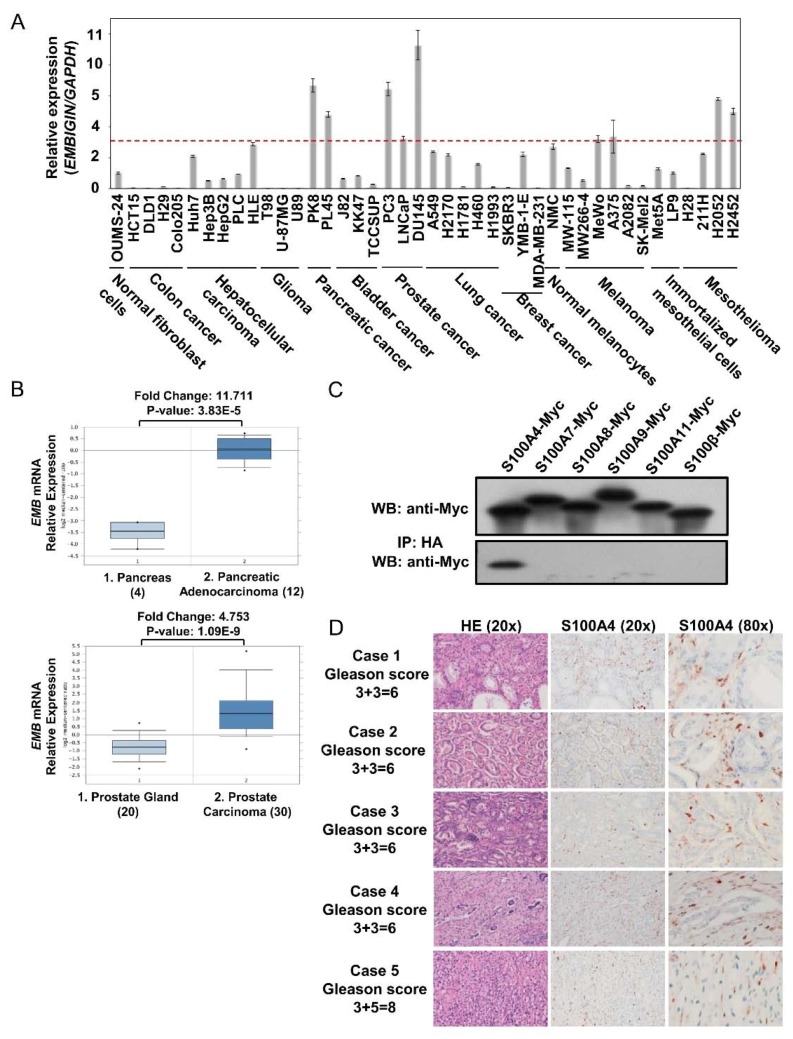
Embigin expression is high in prostate cancer cells and tissues with S100A4 as a novel ligand for embigin. (**A**) The mRNA expression levels of embigin relative to *Tbp* as an internal control gene in various cancer cell lines compared to the expression level in normal fibroblast cells were determined by qRT-PCR. Data are presented as means ± SD. (**B**) Embigin mRNA expression levels in pancreatic adenocarcinoma and prostate carcinoma were significantly higher than the expression levels in the normal pancreas and prostate gland. (**C**) Pull-down assays of HA-tagged embigin co-overexpressed in HEK293T cells with Myc-tagged S100 proteins, S100A4, S100A7, S100A8, S100A9, S100A11 and S100β, showed that S100A4 bound to embigin as detected by WB. (**D**) Immunohistochemistry of S100A4 in tissue samples from prostate cancer patient with Gleason scores of 6–8. S100A4 expression is prominent in the area surrounding the tumor.

In order to understand the importance of embigin in cancer progression, we determined embigin mRNA and protein expression levels in various cancer cell lines by quantitative PCR and WB analysis. We found that pancreatic and prostate cancer cell lines consistently expressed embigin mRNA at levels that were more than 3-fold higher than the level in normal fibroblast cells (Figure 1A). PK-8 cells for pancreatic cancer cells and DU145 cells for prostate cancer cells also produced embigin protein at markedly higher levels (Figure S1A, Supplementary Materials). Moreover, embigin mRNA expression levels in samples from patients with pancreatic adenocarcinoma [14] and prostate carcinoma [15] were significantly higher than the levels in normal pancreas or prostate gland tissues as analyzed via www.oncomine.org (Figure 1B). These data suggest that embigin has an important functional role in pancreatic and prostate cancer progression. Throughout this study, we focused on DU145 cells to clarify the unidentified role of embigin in prostate cancer progression as those cells showed the highest embigin expression at both mRNA and protein levels.

We previously reported that EMMPRIN, ALCAM, and MCAM are receptors for S100A8/A9 [16,17]. Together with RAGE, we proposed these receptors as S100 protein Soil Sensor Receptors (SSSRs). We identified embigin as a paralog of EMMPRIN, which belongs to the immunoglobulin superfamily like SSSRs that induce intracellular signaling by ligand-receptor binding. Therefore, this study aims to identify a specific ligand for embigin and its roles in prostate cancer progression.

Enrichment of S100 proteins in a cancer microenvironment is one of the defining factors for cancer progression. Due to the similarity of embigin to SSSRs, we focused on S100 proteins, which have been reported to be associated with cancer progression. We found by immunoprecipitation that S100A4 is the only S100 protein that binds to embigin and that there is no binding of embigin with S100A8/A9 as there is for SSSRs (Figure 1C). Notably, we also confirmed S100A4 expression in prostate cancer tissue surrounding a tumor with a high Gleason score (6–8) by immunohistochemistry (Figure 1D).

In this study, we evaluated the biological importance of S100A4 binding to embigin by three different approaches: loss-of-function by embigin knockdown and gain-of-function by transient and stable overexpression of embigin. Short interference RNA targeting the embigin gene sequence, reduced embigin endogenous expression by 60–80% for loss-of-function analysis (Figure S1B, Supplementary Materials). For gain-of-function analysis, we used DU145 cells that transiently and stably overexpressed the full length of embigin (wild-type) or embigin cytoplasmic tail (EMB Cyt) (Figure S1C, Supplementary Materials).

### 2.2. S100A4 Binding to Embigin Augments Migration Ability of Prostate Cancer Cells

Extracellular S100A4 has been reported to provide a driving force to cancer cells in the metastatic process [18] by stimulating motility of cancer cells [13,19] and by activating endothelial cells, leading to enhanced angiogenesis [8]. A recent study also showed that embigin positively regulates cellular motility, MMP secretion, and TGF-β downstream signaling in pancreatic cancer [6].

**Figure 2:**
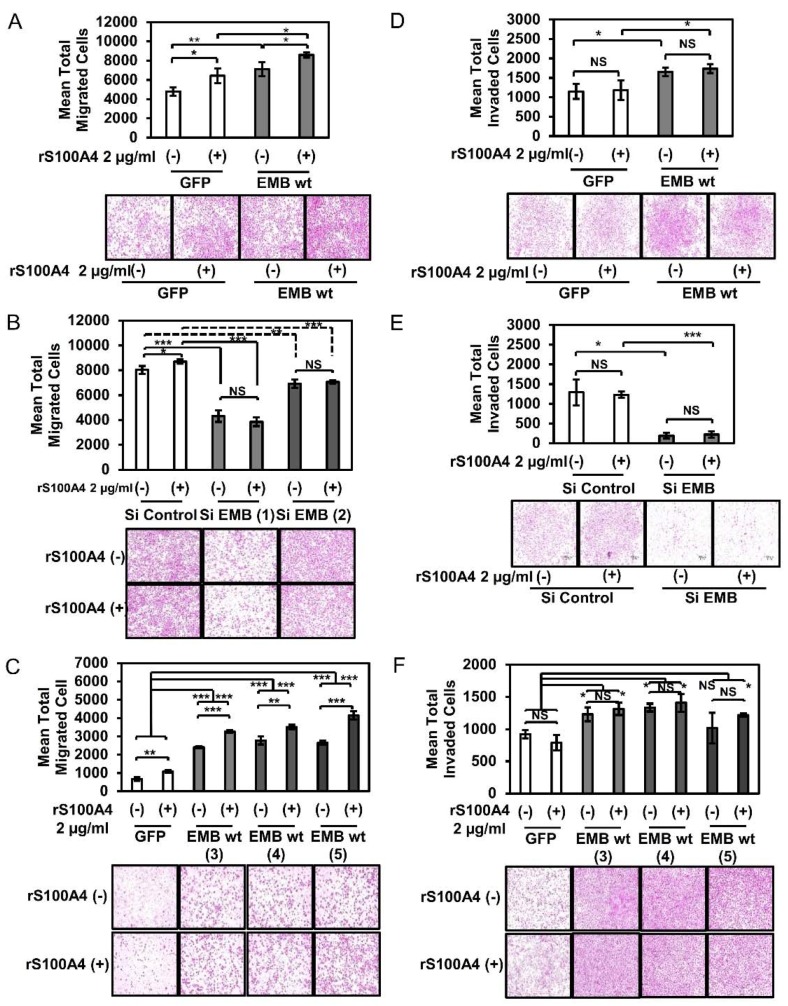
S100A4 binding to embigin augments migration ability of prostate cancer cells. (**A**–**C**) Migration assay of DU145 (**A**) transiently overexpressing GFP and embigin wild-type, (**B**) transfected with siRNA control and two different siRNA targeting *EMB*, (**C**) stably overexpressing GFP and embigin wild-type that were stimulated with 2 µg/mL of rS100A4 for 18 h (*n* = 3). The migration ability of DU145 cells was up-regulated by embigin in an S100A4-dependent manner. (**D**–**F**) Invasion assay of DU145 (**D**) transiently overexpressing GFP and embigin wild-type, (**E**) transfected with siRNA control and siRNA targeting *EMB*, (**F**) stably overexpressing GFP and embigin wild-type that were stimulated with 2 µg/mL of rS100A4 for 18 h (*n* = 3). Embigin up-regulated DU145 cells invasion ability independently to S100A4. One representative image is shown for each group. Data are presented as means ± SD. NS, not significant; * *p* < 0.05; ** *p* < 0.01; *** *p* < 0.001, Student’s *t* test. Experiments were repeated at least twice.

Accordingly, we first evaluated the effect of the S100A4-embigin axis on cancer cell migration. The Boyden chamber assay showed that the migration ability of DU145 cells was remarkably upregulated by an increased level of exogenous embigin and was further enhanced by stimulation with S100A4 (Figure 2A,C). On the other hand, siRNA-mediated knockdown of embigin reduced migration ability even with S100A4 stimulation (Figure 2B). 2 µg/mL of S100A4 was the optimal concentration to induce migration of DU145 cells in our experimental setting (Figure S1D, Supplementary Materials). Unexpectedly, different results in part were obtained in an invasion assay. Embigin mediated a significant increase in the invasion ability of DU145 cells, but treatment with S100A4 did not further enhance invasion ability of the cells (Figure 2D,F). Notably, embigin-mediated invasion of DU145 cells was dramatically reduced by embigin siRNA (Figure 2E) without any appreciable difference with S100A4 stimulation. These results indicate that S100A4 binding to embigin mediates migration of prostate cancer cells. However, S100A4 binding does not contribute to the embigin-mediated invasion of prostate cancer cells.

### 2.3. Embigin Induces NF-κB and MMP9 Activation

Previous studies showed that extracellular S100A4 induces NF-κB activation [8,12] and mediates cancer progression in colorectal cancer, thyroid cancer, malignant melanoma, and prostate cancer [7,19]. Extracellular S100A4 also stimulates matrix metalloproteinase (MMP) production via the RAGE receptor [8,12]. Moreover, S100A4 accelerates tumorigenesis and invasion of human prostate cancer by transcriptional regulation of MMP9 [20]. These studies indicate that NF-κB activation and subsequent regulation of target genes including MMP9 might be involved in S100A4-mediated metastatic progression.

**Figure 3:**
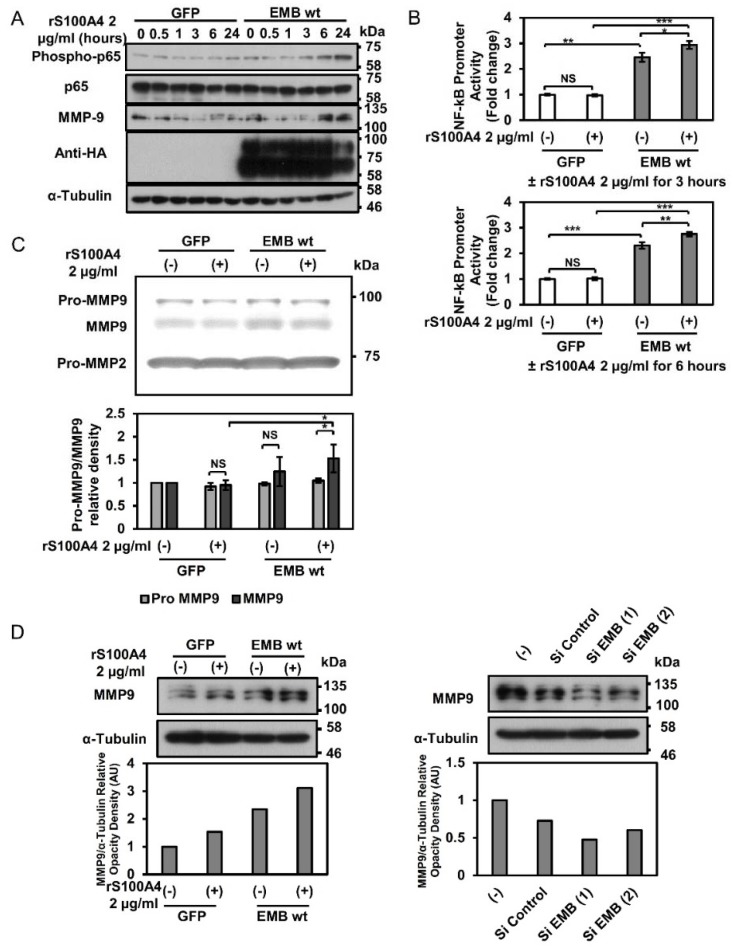
Embigin induces NF-κB and MMP9 activation in prostate cancer cells. (**A**) DU145 cells transiently overexpressing GFP and embigin wild-type were stimulated with 2 µg/mL of rS100A4 at the indicated times and evaluated by WB analysis. Binding of S100A4 to embigin induces NF-κB activation as shown by profound phosphorylation of p65 at 3, 6, and 24 h and higher expression of MMP9 at 6 and 24 h compared to those in GFP-control cells. (**B**) NF-κB promoter activity was evaluated by a luciferase promoter assay in DU145 cells co-transfected with GFP or embigin wild-type and an NF-κB luciferase reporter plasmid and then stimulated with 2 µg/mL of rS100A4 for 3 and 6 h (*n* = 3). Binding of S100A4 to embigin significantly induces NF-κB promoter activity. (**C**) Embigin mediates MMP9 activation as shown by zymography, and inverted band densitometry was quantified by imageJ (*n* = 3). (**D**) MMP9 intracellular protein expression showed a trend in the up-regulation by embigin-dependent to S100A4 as shown by WB analysis, and band densitometry was quantified by imageJ. Data are presented as means ± SD. NS, not significant; * *p* < 0.05; ** *p* < 0.01; *** *p* < 0.001, Student’s *t* test. Experiments were repeated at least twice.

We tried to determine the possible axis of S100A4/embigin-NF-κB-MMP9 in DU145 cells. Western blot analysis showed that S100A4 binding to overexpressed embigin induced NF-κB (p65) phosphorylation and expression of MMP9 in a similar time courses (Figure 3A). A reporter assay using the NF-κB responsive element confirmed that embigin overexpression increased NF-κB promoter activation, which was further induced by S100A4 (Figure 3B). Accordingly, zymography results showed that overexpressed embigin markedly induced MMP9 activation (Figure 3C, upper panel), which S100A4 stimulation slightly increased it (Figure 3C, lower panel). Moreover, WB results showed overexpressed embigin markedly induced MMP9 protein expression, and the induction was slightly upregulated by S100A4 treatment (Figure 3D, left panel). Meanwhile, knockdown of embigin reduced MMP9 protein expression (Figure 3D, right panel). Taken together, these results indicate that embigin plays a critical role in the induction of NF-κB activation and subsequent MMP9 expression with its elevated enzymatic activity. Considering that there was no appreciable induction of NF-κB and MMP9 in GFP-control cells, S100A4-binding might not be mainly contributed on this pathway as invasion ability was independent on S100A4.

### 2.4. Embigin Induces Tumor Growth and Colony- and Spheroid-Forming Ability Independently of S100A4

The secretory S100A4 protein in the stroma has pro-growth effects on prostate cancer cells through NF-κB activation upon RAGE binding [21]. Our results showed that embigin induces prostate cancer cells migration and activation of NF-κB, and MMP9 activation and that the migration was enhanced by extracellular S100A4. This prompted us to disclose another role of embigin in promoting prostate cancer cells in association with extracellular S100A4.

**Figure 4:**
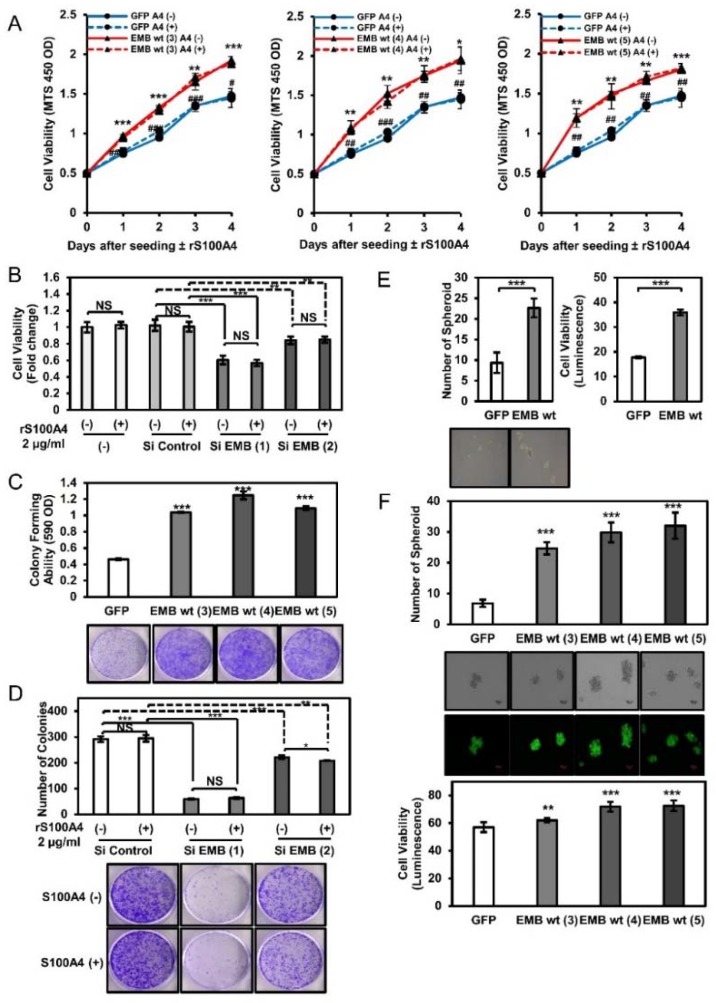
Embigin induces tumor growth and colony- and spheroid-forming ability independently of S100A4. (**A**) MTS assays for 4 days of three different clones of DU145 cells stably overexpressing embigin stimulated by 2 µg/mL of rS100A4 showed an increase in growth rate compared with that of GFP-control cells (*n* = 3). GFP A4 (−) compared to that of EMB A4 (−) NS: not significant; * *p* < 0.05; ** *p* < 0.01; *** *p* < 0.001; GFP A4 (+) compared to that of EMB A4 (+) # *p* < 0.05; ## *p* < 0.01; ### *p* < 0.001, Student’s *t* test. (**B**) MTS assay of DU145 cells with transient knockdown of embigin by siRNA showed a decrease in growth rate compared with the growth rates of non-treated and siRNA control-treated cells stimulated by 2 µg/mLof rS100A4 for 24 h (*n* = 4). These results indicate that embigin induces prostate cancer cell growth independently of S100A4. (**C**) Significant increase in colony-forming ability of DU145 cells stably overexpressing embigin compared with that of GFP-control cells (*n* = 6). (**D**) A colony formation assay of DU145 cells with transient knockdown of embigin by siRNA showed a decrease in the number of colonies compared with that of siRNA control-treated cells stimulated by 2 µg/mL ofrS100A4 for 6 days (*n* = 3). (**E**,**F**) DU145 cells transiently (**E**) or stably (**F**) overexpressing embigin showed significantly increased spheroid-forming ability compared with that of GFP-control cells (**E**, left panel; **F**, upper panel) and showed increased viability as determined by a cell titer-Glo assay (**E**, right panel; **F**, lower panel) after being cultured in a low attachment plate for 6 days (*n* = 6). Data are presented as means ± SD. NS, not significant; * *p* < 0.05; ** *p* < 0.01; *** *p* < 0.001, Student’s *t* test.

We found that overexpressed embigin promoted the proliferation of DU145 cells, while knockdown of embigin suppressed the proliferation of DU145 cells. However, S100A4 binding to embigin did not further induce growth of prostate cancer cells (Figure 4A,B). Additionally, we showed that embigin significantly induced colony formation of DU145 cells stably overexpressing embigin (Figure 4C). Thus, reduced embigin expression by siRNA also suppressed colony formation of DU145 cells independently of extracellular S100A4 (Figure 4D).

One of the multiple stages of cancer progression requires cell survival in the circulation or cell growth in a secondary metastatic site. This stage is partially represented by spheroid-forming ability. Spheroid-forming assay results showed that DU145 cells that transiently or stably overexpressed embigin acquired significantly greater spheroid-forming ability and cell viability than those of GFP-control cells (Figure 4E,F). Taken together, our results indicate that embigin mediates multiple cancer cell behaviors including migration, invasion, growth, colony formation, and spheroid formation. S100A4 binding contributes mainly to the migration process, suggesting that overexpressed embigin in cancer cells and S100A4 in the surrounding stroma (Figure 1D) are coordinately functional for prostate cancer progression.

### 2.5. Intracellular Cytoplasmic Tail of Embigin Is Vital for Prostate Cancer Growth

We next investigated the importance of the embigin cytoplasmic tail for mediating intracellular downstream signals in prostate cancer progression. For this purpose, we constructed plasmid DNA of embigin cytoplasmic domain (EMB Cyt) for transient or stable over-expression in DU145 cells, which highly express endogenous embigin. The overexpressed cytoplasmic tail in DU145 cells competitively binds to intracellular interacting proteins of endogenous embigin cytoplasmic tail, thus inhibits embigin downstream signaling.

**Figure 5:**
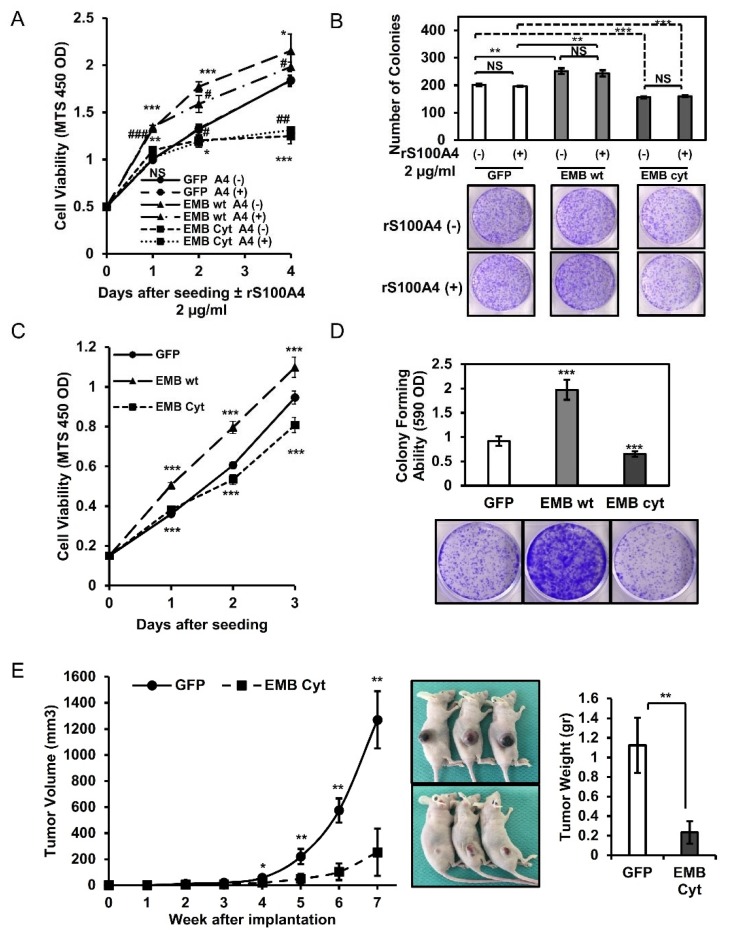
The intracellular cytoplasmic tail of embigin is vital for prostate cancer cell growth in vitro and in vivo. (**A**,**B**) DU145 cells transiently overexpressing GFP, embigin wild-type (wt), and the cytoplasmic tail of embigin (EMB Cyt) stimulated with 2 µg/mL of rS100A4 were evaluated by the MTS assay (**A**) or colony formation assay (**B**) (*n* = 3). (**C**,**D**) DU145 cells stably overexpressing GFP, embigin wild-type (wt), and the cytoplasmic tail of embigin (EMB Cyt) were evaluated by the MTS assay (**C**) or colony formation assay (**D**) (*n* = 6). DU145 cells overexpressing embigin wild-type showed increases in growth rate and colony-forming ability, whereas overexpression of the cytoplasmic tail of embigin inversely suppressed growth rate and colony-forming ability compared with those of GFP-control cells. (**E**) An in vivo growth assay showed significant decreases in the volume (left panel) and weight (right panel) of tumors with DU145 cells stably overexpressing the cytoplasmic tail of embigin compared with those of tumors with GFP-control cells (*n* = 3 mice per group). Data are presented as means ± SD. NS, not significant; * *p* < 0.05; ** *p* < 0.01; *** *p* < 0.001, Student’s *t* test.

Transient or stable overexpression of embigin in DU145 cells increased the growth rate (Figure 5A,C) and colony-forming ability (Figure 5B,D), while overexpression of embigin cytoplasmic tail remarkably reduced the growth rate and colony-forming ability. Furthermore, by using an in vivo growth assay mouse model, we found that the volume and weight of tumor with DU145 cells stably overexpressing the cytoplasmic tail of embigin showed significantly smaller than those of tumor with GFP-control cells (Figure 5E), whereas overexpressed embigin inversely increased tumor volume and weight, and Ki67-positive cells (Figure S2A,B, Supplementary Materials). These results suggest that the cytoplasmic tail of embigin is vital for down-stream signaling that plays a crucial role in prostate cancer progression in vitro and in vivo.

### 2.6. Embigin Mediates Prostate Cancer Cells Survival upon Chemotherapy and Oxidative Stress

Castration-resistant prostate cancer (CRPC) is considered as a progression of disease with docetaxel as the first-line option for treatment. In CRPC, docetaxel induces phosphorylation of bcl-2 (B-cell lymphoma 2), which leads to inactivation of bcl-2, resulting in caspase activation and apoptosis [22]. However, approximately 40–50% of CRPC cases do not respond to treatment with docetaxel [23], suggesting that a docetaxel-resistant cancer cell population appears in a high percentage of CRPC patients.

**Figure 6:**
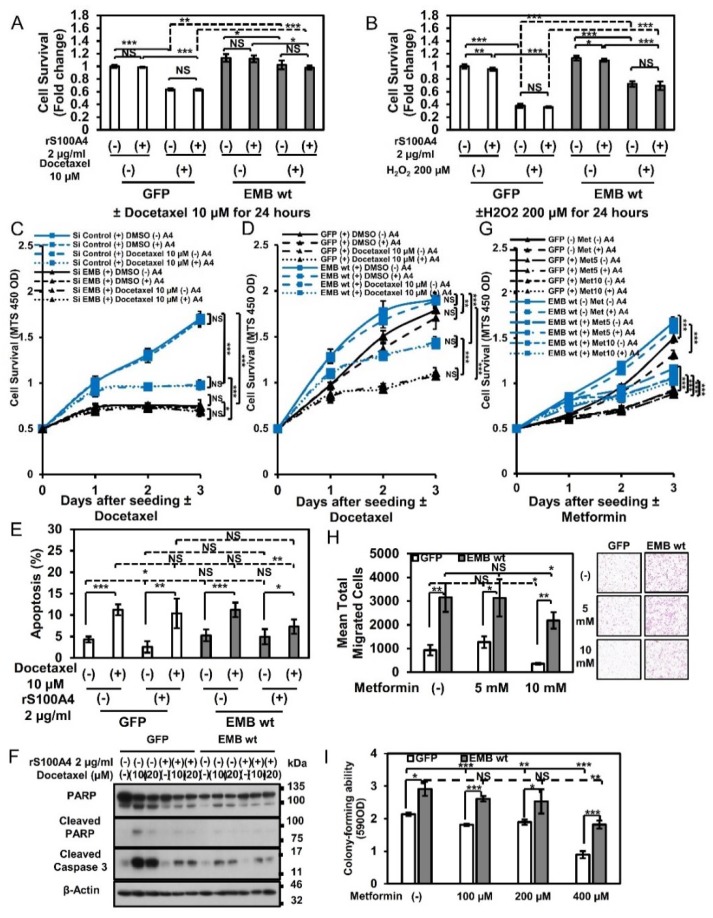
Embigin induces prostate cancer cell survival upon chemotherapy and oxidative stress. (**A**,**B**) DU145 cells transiently overexpressing embigin wild-type (wt) stimulated with or not stimulated with 2 µg/mL of rS100A4 and 10 µM of docetaxel (**A**) or 200 µM of H_2_O_2_ (**B**) showed significantly higher survival rates than those of GFP-control cells as evaluated by the MTS assay (*n* = 3). (**C**) MTS assay of DU145 cells with transient knockdown of embigin by siRNA showed a significant decrease in survival rate compared with that of siRNA control-treated cells stimulated with 10 µM of docetaxel independently of S100A4 (*n* = 8). (**D**) MTS assay showed a marked increase in the survival rate of DU145 cells stably overexpressing embigin compared with that of GFP-control cells stimulated with 10 µM of docetaxel independently of S100A4 (*n* = 8). (**E**) DU145 cells transiently overexpressing embigin wild-type (wt) stimulated with or not stimulated with 2 µg/mL of rS100A4 and 10 µM of docetaxel showed no difference in percentage of late apoptotic cells compared with those of GFP-control cells as determined by Hoechst staining (*n* = 3). (**F**) WB analysis of DU145 cells stably overexpressing GFP and embigin wild-type (wt) stimulated with or not stimulated with 2 µg/mL of rS100A4 and 10 or 20 µM of docetaxel for 24 h. Embigin mediates a reduction in induction of early apoptosis as shown by decreased expression of cleaved PARP and caspase 3. (**G**) MTS assay of DU145 cells stably overexpressing embigin wild-type (wt) that were treated with metformin (5–10 mM) showed a significantly higher survival rate in a dose-dependent manner and independently of S100A4 (*n* = 8) compared with that of GFP-control cells. (**H**) Migration assay of DU145 cells stably overexpressing GFP and embigin wild-type that were treated with 5–10 mM of metformin for 18 h (*n* = 3). DU145 cells overexpressing embigin retained significantly high migration ability under the condition of treatment with 10 mM of metformin compared with that of GFP-control cells. (**I**) Embigin mediated survival in DU145 cells with high colony-forming ability under the condition of continuous treatment with metformin (100–400 µM) for 6 days compared with that of GFP-control cells (*n* = 3). Data are presented as means ± SD. NS, not significant; * *p* < 0.05; ** *p* < 0.01; *** *p* < 0.001, Student’s *t* test.

As shown in Figure 1A, embigin expression is significantly high in DU145 cells, which is one of the androgen-independent prostate cancer cell lines with brain metastasis. We hence hypothesized that embigin plays a critical role in survival of prostate cancer cells during docetaxel treatment. Since production of reactive oxygen species (ROS) is a crucial step for taxane cytotoxicity in vitro and in vivo [24], we evaluated the effects of docetaxel and oxidative stress inducers on embigin-coupled cellular survival. As shown in Figure 6, DU145 cells transiently overexpressing embigin showed increased survival after treatment with docetaxel (Figure 6A) and after treatment with hydrogen peroxide, an oxidative stress inducer (Figure 6B). S100A4 stimulation did not show any appreciable enhancement of the survival mediated by overexpressed embigin. Similar results were also obtained in LNCaP cells, which are an androgen-dependent prostate cancer cells with lymph node metastasis (Supplementary Figure S3A,B). Moreover, embigin mediated survival of DU145 cells treated with increasing concentration of docetaxel when the expression level of embigin was controlled by either siRNA or wild-type stable overexpression (Figure S3C,D, Supplementary Materials), and the survival of cells was not affected by S100A4 stimulation (Figure 6C,D).

The role of apoptotic induction for embigin mediating gain-of-survival after docetaxel treatment was also investigated. Nuclear condensation assessment by Hoechst staining showed no differences between the percentages of late apoptotic cells in GFP-control cells and embigin-overexpressed cells after treatment with docetaxel even at a high concentration (Figure 6E, Figure S3E, Supplementary Materials). However, WB analysis revealed that overexpressed embigin reduced early apoptosis induction as shown by decrease cleaved PARP and caspase 3 expression, which was further decreased by S100A4 stimulation (Figure 6F).

Metformin activity against prostate cancer cell lines has been demonstrated by in vitro and in vivo animal models [25]. Thus, we investigated whether embigin also acts on resistance to the effect of metformin. The results of a cellular viability assay showed that embigin overexpression protects cells from the effect of metformin (1–50 mM) (Figure S3F, Supplementary Materials) and that the cells were not affected by extracellular S100A4 (Figure 6G). Furthermore, metformin at a high concentration (10 mM) significantly decreased migration activity in GFP-control cells, but a high concentration of metformin had little effect on embigin-overexpressed cells (Figure 6H). In addition, embigin-overexpressed cells, but not GFP-control cells, maintained a high level of colony-forming ability in the continuous presence of metformin at 400 µM (Figure 6I). Similar results were also obtained for cells treated with rapamycin, an mTORC1 inhibitor, by which embigin mediated higher cell survival (Figure S4A, Supplementary Materials) and maintained high migration ability in the presence of S100A4 (Figure S4B, Supplementary Materials). These results indicate that embigin contributes greatly to the chemo-resistance of prostate cancer cells.

### 2.7. Binding of S100A4 to Embigin Mediates its Biological Functions through AMPK/mTOR/p21 Signaling and Inhibits Autophagy

AMP-activated protein kinase (AMPK) is a critical sensor of cellular energy and nutrient levels. Loss of AMPK or deregulation of its activity has been shown to be linked tightly to cancer development and progression [26]. We hence examined whether AMPK has a suppressive function in the embigin downstream signaling pathway.

**Figure 7:**
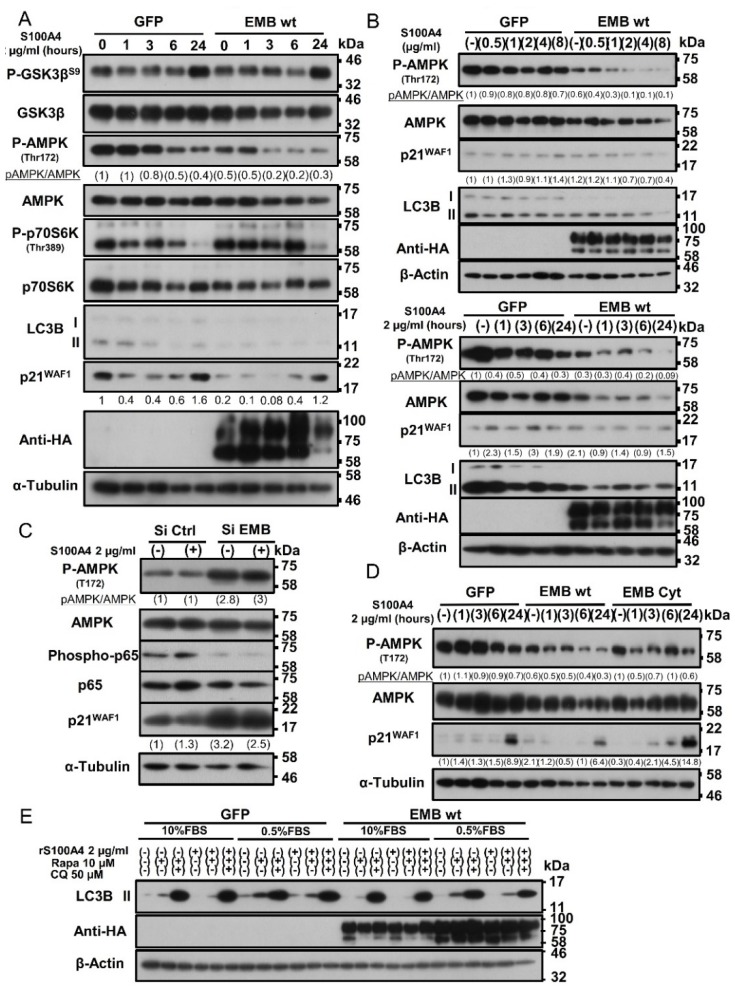
Embigin mediates its biological functions through AMPK/mTOR/p21 signaling and inhibition of autophagy. (**A**–**D**) Western blot analysis of (**A**) DU145 cells transiently overexpressing GFP and embigin wild-type (wt) that were stimulated with 2 µg/mL of rS100A4 for 1, 3, 6, and 24 h. Embigin activates mTORC1 signaling as indicated by higher phosphorylation of p70S6K, followed by inhibition of the cell cycle inhibitor p21 and the autophagy marker LC3B-II. (**B**) DU145 cells stably overexpressing GFP and embigin wild-type (wt) were stimulated with increasing concentrations of rS100A4 (0.5–8 µg/mL) for 6 h (upper panel) or with 2 µg/mL of rS100A4 for 1, 3, 6 and 24 h (lower panel). Embigin induces dephosphorylation of AMPK in a dose- and time-dependent manner. (**C**) DU145 cells with transient knockdown of embigin by siRNA and siRNA control-treated cells were stimulated with 2 µg/mL of rS100A4 for 24 h. Knockdown of embigin oppositely increases AMPK phosphorylation and p21 expression. (**D**) DU145 cells stably overexpressing GFP, embigin wild-type (wt), and the cytoplasmic tail of embigin (EMB Cyt) were stimulated with 2 µg/mL of rS100A4 for 1, 3, 6 and 24 h. The cytoplasmic tail of embigin abrogates AMPK dephosphorylation and induces higher expression of p21. (**E**) Western blot analysis of DU145 cells stably overexpressing GFP and embigin wild-type (wt) that were cultured in 10% or 0.5% FBS-supplemented medium for 24 h after seeding and then stimulated with 2 µg/mL of rS100A4 for 3 h prior to treatment with DMSO, rapamycin (10 µM) and chloroquine (50 µM) for 5 h.

Interestingly, as shown in Figure 7A, we found that binding of S100A4 to embigin induced marked dephosphorylation of AMPK in a time-dependent manner in cells with transiently overexpressed embigin compared to that in GFP-control cells. This was further confirmed by DU145 cells stably overexpressed embigin that showed a dose-and time-dependency of S100A4 to dephosphorylate AMPK (Figure 7B). Numerous studies have revealed that the mTOR pathway is one of the major growth regulatory pathways controlled by AMPK [26]. Thus, we focused on molecules related to mTOR signaling. Phosphorylation of p70S6K on Thr389 is considered to be a reliable marker for detecting activation of mTORC1 signaling [27]. In accordance with the dephosphorylation of AMPK, there was an increased level of p70S6K phosphorylation, which was more obvious in embigin-overexpressed cells than in GFP-control cells (Figure 7A).

AMPK activation has been shown to inhibit cell proliferation through multiple mechanisms including p53 stabilization [28] and regulation of the cyclin dependent kinase (CDK) inhibitors p21^WAF1^ and p27^Kip1^ [29,30]. Expression of the cell cycle inhibitor p21^WAF1^ was significantly suppressed by rS100A4 stimulation in GFP-control cells for up to 6 h. Cells with embigin overexpression showed greater suppression of p21^WAF1^ expression during rS100A4 treatment for 6 h (Figure 7A). Since DU145 is a p53-mutated cell line, the results suggested that growth induction through the S100A4-embigin/AMPK/mTORC1/p21 axis is independent of p53. In addition, the appearance of LC3B-II, an essential component to induce autophagy, was mitigated after treatment of control cells with rS100A4. This was further suppressed in embigin-overexpressed cells (Figure 7A). Almost the same patterns of molecular signaling were also observed in both the LNCaP prostate cancer cell line in a transient expression system (Figure S5A, Supplementary Materials) and the DU145 subline with stable expression of exogenous embigin (Figure 7B). In contrast, knockdown of endogenous embigin resulted in a high phosphorylation level of AMPK and increased level of p21^WAF1^ in DU145 cells (Figure 7C).

Aggressive cancer cell lines can also activate AMPK-independent signaling pathways under the condition of metabolic stress induced by serum starvation, including the signaling pathways mediated by Akt and ERKs, as part of the metabolic adaptation to hostile environments [31]. Accordingly, rS100A4 stimulation within 24 h under the condition of serum starvation, increased ERK and JNK phosphorylation at comparable levels in both GFP-control cells and embigin-overexpressed cells. Notably, under this condition, embigin-overexpressed cells showed markedly decreased phosphorylation of Akt and p38 along with AMPK dephosphorylation compared to those in GFP-control cells as shown by WB results (Figure S5B, Supplementary Materials). Interestingly, after 30 min of rS100A4 stimulation, embigin mediated significant dephosphorylation of AMPK, Akt, ERK, p38, and JNK compared to that in GFP-control cells (Figure S5C, Supplementary Materials). These results suggest that S100A4-embigin-mediated prostate cancer progression is independent of Akt and MAPK signaling.

As shown in Figure 5, the intracellular cytoplasmic tail of embigin is vital for prostate cancer cell proliferation. We observed that DU145 cells stably overexpressing the embigin cytoplasmic tail showed reduced activity for dephosphorylation of AMPK that was linked to higher expression of p21^WAF1^ in vitro and in vivo (Figure 7D, Figure S2C, Supplementary Materials). AMPK also regulates autophagy directly or indirectly via mTORC1 [26]. In the present study, binding of rS100A4 to embigin suppressed the appearance of LC3B-II under both metabolic stress-plus and -minus condition (0.5% or 10% FBS supplemented medium) or under the condition of treatment with rapamycin, a strong autophagy inducer, suggesting that the S100A4-embigin signal(s) exerts autophagy inhibition (Figure 7E). These results suggest that the S100A4-embigin axis mediates prostate cancer progression through AMPK suppression, which activates mTORC1 leading to suppression of p21^WAF1^ expression, which results in the induction of proliferation and inhibition of autophagy.

### 2.8. Embigin and p21^WAF1^ Expression in Prostate Cancer Tissue can Predict Survival

**Figure 8:**
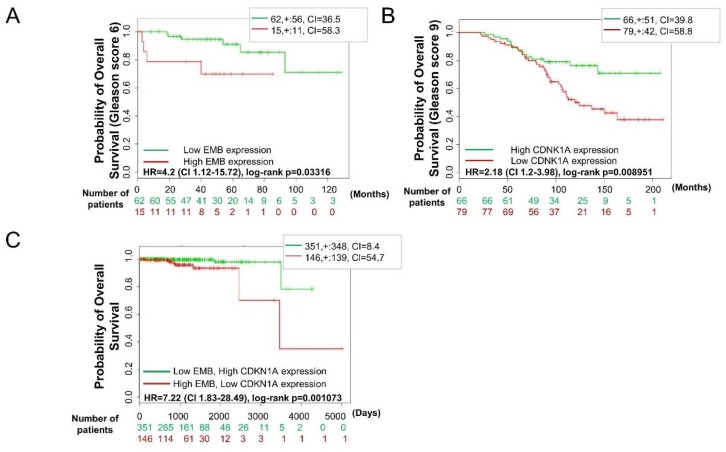
Embigin and p21 (CDKN1A) expression in prostate cancer tissue can predict survival. (**A**) Overall survival determined by Kaplan-Meier analysis of prostate cancer patients with either high or low *EMB* mRNA expression, (**B**) high or low *p21 (CDNK1A)* mRNA expression, and (**C**) high *EMB* and low *p21 (CDNK1A)* mRNA expression or low *EMB* and high *p21 (CDNK1A)* mRNA expression.

We further investigated the clinical relevance of embigin and its downstream-mediated signaling in prostate cancer progression by using SurvExpress [32]. With publicly available data sets, we found expression profiling of high Embigin (gene name: *EMB*) or low p21^WAF1^ (gene name: *CDKN1A*) mRNA in prostate cancer patients [33,34], which leads to worse overall survival (Figure 8A,B). This result was more prominent in prostate cancer patients with a higher Gleason score. Moreover, data generated by the TCGA Research Network (http://cancergenome.nih.gov/) revealed significantly worse survival in prostate cancer patients regardless of the Gleason score. The pattern was high *EMB* and low *CDKN1A* mRNA expression in prostate cancer tissues (Figure 8C). These data suggest that embigin and p21^WAF1^ are promising biomarkers for predicting overall survival of prostate cancer patients.

## 3. Discussion

Embigin has been reported to associate with membrane monocarboxylate transporters 2 (MCT2), which transports monocarboxylates across the plasma membrane [35]. Recent study showed MCT2 protein overexpression promotes the growth of prostate cancers by epigenetic regulation of MCT2 isoforms and identifies a link between MCT2, Androgen Receptor (AR), ETS-related gene (ERG) and other oncogenic pathways in prostate cancers [36]. Additionally, peroxisomal MCT2 has reported to increase in β-oxidation levels, which may be crucial for malignant transformation of prostate cancers [37]. Notably, other immunoglobulin superfamily member, CD147 has also associated with MCT1 and MCT4 that required for maintaining the catalytic activity of MCTs and their translocation to the plasma membrane [35], which leads to control the energetics of glycolytic tumors [38]. Furthermore, the genetic approach that disrupted the MCT/CD147 complexes alone or in combination with phenformin inhibits tumor growth in vitro and in vivo [39]. This suggests embigin might affect prostate cancer growth by its interaction with either MCT2 or other MCTs that reprogram cancer metabolic activity independently to S100A4. In addition, recently Dange et al. succeeded to identify another embigin ligand Galectin-3 as a potential candidate by utilizing mass spectrometry based identification strategy [40]. The interaction also contributed to an acquisition of cancer metastatic properties. Thus, embigin may have several ligands including membrane proteins like MCTs and extracellular soluble proteins such as S100A4 and Galectin-3.

AMPK is a well-known energy sensor kinase [41], however, its role in cancer progression has remain an enigma with both increase and decrease of AMPK activity have been reported to contribute in cancer progression. Recently, number of studies also revealed that AMPK activation regulates directional cell migration by controlling microtubule dynamics [42], regulation of actin cytoskeleton dynamics and reorganization at the plasma membrane [43]. In contrary, inhibition of AMPK activity also reported to increase the cell surface abundance of β1-integrin, which is a key protein for cell migration, by altering its endomembrane traffic [44]. Additionally, AMPK inhibition mediates cell migration by lamellipodia formation through activation of the Rac1-Arp2/3 signaling pathway [45]. In the present study, we found that S100A4 binding to embigin mediate prostate cancer migration partly through down-regulation of AMPK activity. Further studies required to elaborate the specific migration-related signaling induced by S100A4 binding to embigin.

It has been reported that AMPK can directly elicit changes in transcription through phosphorylation of the transcription factor FOXO3, which regulates EMT in cancer cells through an Akt-dependent mechanism [46], or the core histone H2B in a direct transcriptional and chromatin regulatory pathway leading to cellular adaptation [47] during chronic nutrient starvation. Both phosphorylation of FOXO3 and that of the core histone H2B regulate p21 gene expression in p53-independent and-dependent manner, respectively. However, qPCR analysis showed no significant differences in the expression of EMT-related genes and transcription factors or chemoresistance-associated ABC-transporter genes in embigin-overexpressed cells (Figure S6A,B, Supplementary Materials). Further studies are needed to determine whether binding of S100A4 to embigin induces AMPK dephosphorylation may exert its biological effects by directly modulating phosphorylation of AMPK-targeted substrate transcription factors.

mTOR is aberrantly activated in up to 80% of human cancers, and it up-regulates the translation machinery of proteins that are important for cancer cell growth, survival, invasion, and metastasis [48]. Phosphorylation of mTOR at serine 2448 has been reported as a marker of mTOR activation [27]. However, this site appears to be phosphorylated in both mTORC1 and mTORC2 [49] with unknown molecular functions. Moreover, one study using immunohistochemistry of a large tissue microarray strongly suggested that loss of pSer2448-mTOR expression is a marker for activated AKT/mTOR signaling [50]. In agreement with that study, we found that S100A4-embigin reduced phosphorylation of mTOR at serine 2448 characterized mTOR activation (Supplementary Figure S6C). Additionally, a study by Müller et al. showed that a small (4%) but clinically high relevant fraction of prostate cancers characterized by loss of pSer2448-mTOR expression, ERG fusion and PTEN deletion represents a molecularly distinct subgroup of tumors with aggressive and early recurrent features. In our study, DU145 cells overexpressing embigin, which have been reported as TMPRSS2–ETS fusion-negative cells [51] with PTEN mutation, showed higher mobility and promoted growth and survival. These results suggest that highly progressive prostate cancer could be characterized by reduced mTOR phosphorylation at serine 2448 as a marker of mTOR activation with embigin as a distinctive molecular signature regardless of its genetic oncogenic background.

As is the AMPK/mTOR pathway, IKK/NF-κB signaling is constitutively active in prostate cancer due to dysregulation/activation of oncogenic pathways such as Akt or loss of PTEN tumor-suppressor function [52]. A previous study showed that mTORC1 is involved in Akt-mediated activation of IKK/NF-κB in PTEN loss-induced prostate cancer [53]. In addition, it has been shown that tuberous sclerosis 2 negatively or positively regulates IKK/NF-κB activity in a genetic background-dependent manner [54]. In our study, it was shown that binding of S100A4 to embigin induces NF-κB and mTORC1 activation but reduces Akt activation in a time-dependent manner (Figure S5B,C, Supplementary Materials).

To determine other possible functions of embigin that are relevant to prostate cancer aggressiveness, we performed comprehensive gene expression analysis of DU145 cells stably overexpressing embigin in comparison to GFP-control cells by using an RNA-sequencing technique (Figure S7, Supplementary Materials). By this approach, we found that highest number of genes altered (up and down) in embigin overexpression are associated with cancer diseases (Figure S7A, Supplementary Materials). Most of the genes upregulated by embigin overexpression are associated with cell adhesion and extracellular matrix organization (Figure S7B, Supplementary Materials). This partially explains the enhancement of cellular migration and invasion by embigin (Figure 2). Additionally, embigin-NF-κB/MMP9 signaling may be associated with extracellular matrix organization. Interestingly, ABCA1 and ETV1 are upregulated genes by embigin overexpression in both cancer and metabolic disease cluster (Figure S7C, Supplementary Materials). ABCA1, an ABC transporter, but not ABCB1, ABCC1 or ABCC2 (Figure S7B, Supplementary Materials), might be involved in the anti-cancer drug resistance observed in DU145 cells stably overexpressing embigin (Figure 6, Figure S3, Supplementary Materials). Accumulating evidence indicates a role of transcription factor ETV1 in prostate cancer tumorigenesis and progression [55,56]. However, it is still not known how embigin regulates these cancer-related molecules and whether embigin-mediated AMPK/mTORC1 and NF-κB pathways are required for the regulation of these molecules. In our ongoing study, we will try to determine the mechanism of the signal pathways at the molecular levels.

## 4. Materials and Methods

### 4.1. Cell Lines and Chemicals

Human prostate cancer cell lines DU145 and LNCaP were purchased from American Type Culture Collection (Rockville, MD, USA). Both cancer cell lines were used within five passages and cultured in RPMI medium (Invitrogen, Carlsbad, CA, USA) supplemented with 10% heat-inactivated fetal bovine serum (FBS; Intergen, Purchase, NY, USA) at 37 °C in a humidified atmosphere containing 5% CO_2_. DU145 stably-overexpressing GFP, embigin wild-type (EMB wt), and cytoplasmic tail of embigin (EMB Cyt) cells were cultured in a medium supplemented with 10 µg/mL puromycin (EMD Biosciences, La Jolla, CA, USA) and 10% FBS. Docetaxel and rapamycin were purchased from TCI (Tokyo Chemical Industry, Tokyo, Japan) and 1.1-dimethylbiguanide hidrochloride (Metformin) was purchased from Sigma Aldrich (St. Louis, MO, USA). The antibodies used in this study are listed in Supplementary Table S1.

### 4.2. Preparation of S100A4 Protein

BL21 competent cells (TAKARA, Shiga, Japan) were transformed with the pGEX-6P-3-S100A4 plasmid and then cultured overnight in 10 mL Luria-Bertani (LB) medium (Sigma-Aldrich, St. Louis, MO, USA) supplemented with 100 µg/mL of Ampicillin and subsequently transferred into large culture in 100 mL LB medium supplemented with 100 µg/mL of carbenicillin for 3–6 h using an orbital shaker (125 rpm) at 37 °C. When optical density (OD)600 reached 0.5, 200 µL 1M isopropyl β-d-1-thiogalactopyranoside (final concentration of 2 mM) was added to induce protein synthesis, and the culture was continued for 6 h at room temperature (22–25 °C) with gentle shaking. Transformed BL21 cells were collected by centrifugation and suspended in 10 mL phosphate buffer solution (PBS) and then stored at −80 °C following purification.

For purification, a 4-times volume of sonication buffer (1% Triton-X, 50 mM Tris-HCl, 150 mM NaCl, 5 mM DTT, 5 mM EDTA, pH 7.4) was added to PBS-suspended transformed cells for sonication followed by centrifugation at 13,000 rpm for 20 min. The supernatant containing GST-tagged recombinant S100A4 (rS100A4) was mixed with 2 mL Glutathione sepharose 4B beads (GE Healthcare, Piscataway, NJ, USA) and rotated overnight at 4 °C. The sepharose beads were collected into a column and washed repeatedly with ice-cold PBST. Bound rS100A4 on 2 mL beads was eluted with 4 mL elution buffer (20 mM reduced glutathione, 100 mM Tris-HCl, 200 mM NaCl, pH 8.8) for 10 min at 4 °C. Eluted GST-tagged S100A4 was collected and its concentration was measured by the Bradford assay. The total volume of GST-tagged S100A4 was reduced to 3 mL by centrifugation with Amicon ultra-10 (Merck Millipore, Darmstadt, Germany). Subsequently, GST-tagged S100A4 was dialyzed with 2 L of dialysis buffer (50 mM Tris-HCl, 150 mM NaCl, 1 mM EDTA, 1 mM DTT, pH 7.5) using a Slide-A-lyser cassette with continuous stirring at 4 °C. Finally, dialyzed GST-tagged S100A4 was added with PreScission protease (GE Healthcare) overnight at 4 °C to cleave the GST tag. After GST tag cleavage, rS100A4 was dialyzed with 20 mM HEPES and 150 mM NaCl (pH 7.4) at 4 °C and then sterilized with 0.22-μm Millex-GV syringe filters (Merck Millipore). The sterilized rS100A4 was aliquoted and stored at −80 °C until use for biological experiments. The purity of rS100A4 was confirmed by Coomassie Brilliant Blue (CBB) staining after sodium dodecyl sulfate polyacrylamide gel electrophoresis (SDS-PAGE).

### 4.3. Preparation of a Plasmid Vector

GFP cDNA and human cDNAs encoding full-length (wild) embigin (EMB wt) and the embigin cytoplasmic tail (EMB Cyt) were inserted into the newly constructed expression vector pIDT-SMART(C-TSC) [57]. For the establishment of stable clones, GFP, full-length (wild) embigin and embigin cytoplasmic tail (EMB Cyt) cDNAs were inserted into an improved plasmid construct based on the pIDT-SMART (C-TSC) vector. DU145 cells were subjected to transfection with a combination of the prepared plasmids and another vector containing the puromycin-resistance gene. Established clones were selected with 10 µg/mL of puromycin and confirmed by Western blot analysis.

### 4.4. Embigin Knockdown Experiment with Short Interference RNA (siRNA)

Twenty-four hours after seeding 2 × 10^5^ DU145 cells on a 6-well plate, the first transfection of siRNA targeting Embigin (Table S2, Supplementary Materials) or scramble (Ambion/Thermo Fisher Scientific, Waltham, MA, USA) at a final concentration of 25 nM with Lipofectamine RNAiMAX (Invitrogen, Carlsbad, CA, USA) was performed according to the manufacturer’s recommended protocol. At 24 h after transfection, the cells were starved in 0.5% FBS-supplemented medium overnight followed by a second transfection. The cells were then subjected to starvation in 0.5% FBS-supplemented medium overnight prior to stimulation with 2 µg/mL of rS100A4 for the indicated time in each assay.

### 4.5. Embigin-S100 Proteins Binding Assays

Full-length HA-tagged embigin and Myc-tagged S100A4, S100A7, S100A8, S100A9, S100A11, and S100β were purified from cell lysates of transfected HEK293T cells. Subsequently, HA-tagged embigin was incubated with monoclonal anti-HA tag (clone HA-7) agarose beads (Sigma-Aldrich, St Louis, MO, USA) in the presence of Myc-tagged recombinant S100 proteins (2.0 μM) for one hour at 4 °C by rotation. After incubation, bound proteins were pulled-down by centrifugation. The precipitated proteins were subjected to SDS-PAGE and detected by western blotting using a mouse anti-Myc tag antibody (clone 9B11) for bound S100 protein.

### 4.6. Cell Migration and Invasion Assays

DU145 cells transiently or stably overexpressing GFP or embigin wild-type were starved by 0.5% FBS-supplemented medium overnight prior to a migration or an invasion assay. The invasion assay was performed by using transwell culture inserts with 8-μm pore filters (BD Biosciences, Bedford, MA) coated with BD Matrigel™ Basement Membrane Matrix (BD Biosciences) in a 24-well plate. After counting the number of cells by the Trypan Blue exclusion method, 3 × 10^4^–5 × 10^4^ cells in 200 μL 0.5% FBS-supplemented medium were seeded in the upper chamber, and 600 μL of 10% FBS-supplemented medium with or without 2 µg/mL of rS100A4 was added to the lower chamber. The cells were incubated for 18 h at 37 °C. After being fixed with methanol and stained with hematoxylin eosin (Muto Pure Chemicals, Tokyo, Japan), cells on the upper side of the transwell were removed vigorously with a cotton bud. Experiments were carried out in triplicate with five pictures for each transwell being taken by using the all-in-one Fluorescence Microscope BZ-X710 series (Keyence, Osaka, Japan) for quantification. All experiments were repeated at least twice.

### 4.7. Luciferase Reporter Assay 

DU145 cells were transiently co-transfected with NF-κB luciferase reporter and GFP or embigin wild-type plasmid using Lipofectamine^R^2000 according to the manufacturer’s recommended protocol. At 24 h after transfection, 4 × 10^5^ cells were reseeded on a 12-well plate and starved in 0.5% FBS-supplemented medium overnight, and then 2 µg/mL rS100A4 was added for three hours and six hours. Luciferase assays were performed using the britelite^TM^ plus Reporter Gene Assay System (Perkin Elmer, Billerica, MA, USA). The luminescence was determined using a Fluoroskan Ascent FL luminometer (Thermo Fisher Scientific). Experiments were carried out in triplicate.

### 4.8. Zymography 

DU145 cells transiently overexpressing GFP or embigin wild-type were reseeded at 1 × 10^6^ cells on a 60-mm dish at 24 h after transfection and were starved in 0.5% FBS-supplemented medium overnight. The medium was refreshed with serum-free OPTI-MEM (Invitrogen), and 2 µg/mL of S100A4 was added for 48 h. The conditioned medium was collected and concentrated with Amicon Ultra-4 (Merck Millipore) to 100 µL and then an equal volume of non-reducing 2 times SDS sample buffer was added. An equal volume of each sample was loaded in 10% gel with 2 mg/mL gelatin. Subsequently, the gel was washed with a refolding buffer (50 mM Tris-HCl, 100 mM NaCl, 10 mM CaCl_2_, 2.5% Triton-X 100, 10% glycerol, pH 7.4) for 4 h and then incubated in a substrate buffer (50 mM Tris-HCl, 10 mM CaCl2, pH 7.4) at 37 °C for 24 h. The gel was subjected to CBB staining and carefully washed until a clear band appeared on a blue background. The clear band as a sign of MMP activity was scanned and the image was inverted for density quantification by imageJ. Experiments were carried out in triplicate.

### 4.9. RNA Extraction and Real-Time Quantitative Reverse Transcription (qRT)-PCR Analysis

DU145 or LNCaP cells transiently overexpressing GFP or embigin wild-type (wt) were harvested after being stimulated with or not stimulated with 2 µg/mL of S100A4 for 24 h. RNA extraction and Real-time PCR were performed as described previously [16] with specific primers listed in Table S3, Supplementary Materials targeting EMT-related transcription factors or ABC-transporter genes, and TBP on a LightCycler 480 system II (Roche, Mannheim, Germany). The levels of mRNA expression were expressed relative to TBP as an internal control using the ∆∆Ct method.

### 4.10. Cell Viability Assay (MTS Assay)

Two thousand to five thousand DU145 cells transiently or stably overexpressing GFP or embigin wild-type were cultured overnight in a medium supplemented with 0.5% FBS on 96-well plate. Then 2 µg/mL of rS100A4 was added to the FBS-supplemented medium for 24 h followed by treatment with docetaxel, H_2_O_2_ or metformin at the indicated time points. Experiments were carried out at least in triplicate, and cell viability was analyzed by the CellTiter 96^®^ AQueous One Solution Cell Proliferation Assay (Promega BioSciences, San Luis Obispo, CA, USA) according to the manufacturer’s recommended protocol. The absorbance was determined at 450 OD using an iMark^TM^ Microplate Absorbance Reader (Bio-Rad, Hercules, CA, USA). Experiments were repeated at least twice.

### 4.11. Colony Formation Assay

Two thousand DU145 cells transiently or stably overexpressing GFP, embigin wild-type (EMB wt) or embigin cytoplasmic tail (EMB Cyt) were cultured on a 6-well plate with 10% FBS-supplemented medium overnight after seeding. Then 2 µg/mL of rS100A4 was added and the medium was refreshed with 10% FBS-supplemented medium and 2 µg/mL of rS100A4 every 48 h. Seven days after seeding, colonies were fixed with ice-cold methanol for 5 min and then stained with 0.1% crystal violet in PBS for 30 min at room temperature. Then the plates were washed 3 or 4 times with PBS and air-dried. The colony number was quantified either by counting or by destaining with 10% acetic acid in PBS, with absorbance subsequently measured at 590 OD using an iMark^TM^ Microplate Absorbance Reader (Bio-Rad). Experiments were carried out at least in triplicate and repeated at least twice.

### 4.12. Spheroid Formation Assay

One hundred thousand DU145 cells transiently or stably overexpressing GFP or embigin wild-type (EMB wt) were cultured in a serum-free medium supplemented with 20 ng/mL EGF, 20 ng/mL bFGF, 4 µg/mL insulin, B27, N2, and stem protein on a low attachment 6-well plate (Corning, Steuben, NY, USA) for 7 days. Experiments were carried out in hexaplicate with 5 pictures for each well taken using the all-in-one Fluorescence Microscope BZ-X710 series (Keyence, Osaka, Japan). The spheroid number was quantified by counting spheroids with diameters >50 µm. After counting, all of the cells in the medium were collected and subjected to centrifugation at 2500 rpm for 2 min. The cell pellet was suspended in 100 µL serum-free medium. Overall viability of spheroids was analyzed by the *Cell-Titer-Glo*™ Luminescent Cell Viability Assay (Promega) according to the manufacturer’s recommended protocol.

### 4.13. Cell Apoptosis Assay 

DU145 cells transiently overexpressing GFP or embigin wild-type (EMB wt) were cultured in a 0.5% FBS-supplemented medium overnight after seeding. Then 2 µg/mL rS100A4 was added to the FBS-supplemented medium for 24 h followed by treatment with docetaxel or H_2_O_2_ for 24 h. The percentage of apoptotic cells was determined by Hoechst 33342 staining (1 µg/mL). Three pictures for each well were taken at a 200x magnification using an inverted fluorescence microscope (BX50, Olympus, Tokyo, Japan). Experiments were carried out in triplicate and repeated twice.

### 4.14. Western Blotting 

DU145 cells transiently or stably overexpressing GFP, embigin wild-type (EMB wt) or embigin cytoplasmic tail (EMB Cyt) were starved in 0.5% FBS-supplemented medium overnight followed by stimulation with 2 µg/mL of rS100A4 at the indicated time points. The cells were then harvested and subjected to SDS-PAGE and Western blotting (WB) under conventional conditions described previously [16].

### 4.15. Immunohistochemistry

Immunohistochemistry of human prostate cancer tissues and mouse tumor tissue were carried out as described previously [16] with rabbit monoclonal anti-S100A4 (Abcam, Cambridge, UK) or mouse monoclonal anti-Ki67 (DAKO, Santa Clara, CA, USA) used as primary antibodies respectively. The study using the patient-derived tissues was approved by the research ethics committees of Niigata University Medical and Dental Hospital. Informed consent was obtained from each patient for use of the materials.

### 4.16. In vivo Growth Assay

Our animal study was approved by the research ethics committees of Okayama University and performed according to the Institutional Animal Care and Use Committee (IACUC) at Okayama University. DU145 cells stably overexpressing embigin wild-type (EMB), embigin cytoplasmic tail (EMB Cyt) or GFP were subcutaneously injected into the right flank region of 7-week-old BALB/c-nude mice (1 × 10^6^ cells in 0.1 mL PBS/mouse). Tumor size was measured every week by using digital calipers (Mitutoyo, Kanagawa, Japan), and tumor volume was calculated by (smallest diameter)^2^ × largest diameter × 0.5. Seven weeks after tumor implantation, mice were sacrificed and their tumor weights were measured by a digital electronic scale (Sartorius, Gottingen, Germany). We used anesthetic inhalation to avoid suffering of the animal prior to the procedures of tumor injection, tumor size measurement, and sacrifice at the end of observation.

### 4.17. Western Blot Analysis of Mouse Tumors 

Mouse tumors implanted with DU145 cells stably overexpressing GFP or embigin cytoplasmic tail (EMB Cyt) for 7 weeks were immediately stored at −80 °C in a Tissue-Tek (Sakura Finetek Inc., Torrance, CA, USA) after being washed with PBS. Ten 10-µm-thick slices of frozen tissue cut by a microtome (MICROM HM 525, MICROM International GmbH, Walldorf, Germany) were lysed in M-PER (Thermo Fisher) lysis buffer supplemented with PHOSTOP and cOmplete tablet Mini (Roche, Mannheim, Germany) by a homogenizer (Ultra-Turrax T8, IKA Labortechnk, Germany). Ten micrograms of protein were then subjected to SDS-PAGE and subsequent WB under conventional conditions described previously [16].

### 4.18. Statistical Analysis

Data were analyzed by Student’s t-test (two-tailed distribution with two-sample equal variance) using Excel Office 2010 (Microsoft, Tokyo, Japan) and expressed as means ± SD. *p* < 0.05 was considered significant.

## 5. Conclusions

We have shown that embigin contributes to the progression of prostate cancer with multiple cancer behaviors through AMPK/mTORC1/p21^WAF1^ and NF-κB/MMP9 signaling. S100A4 binding to embigin spesifically augments prostate cancer sell migration, which partly due to inhibition of AMPK activity. Many studies have used different strategies to directly target AMPK activity in cancer. However, the pivotal roles of AMPK in numerous physiological processes might limit its use as a therapeutic target. We propose that the S100A4-embigin/AMPK/mTORC1/p21^WAF1^ and NF-κB/MMP9 axis is a vital oncogenic molecular machinery exploited by a certain fraction of prostate cancers for progression. Therefore, targeting S100A4/embigin-mediated signaling is a potential prostate cancer therapeutic approach for prostate cancer patients. Additionally, as our data have shown, embigin and p21^WAF1^ can be used as prognostic biomarkers for prostate cancer patients.

## Supplementary Materials

The following are available online at http://www.mdpi.com/2072-6694/10/7/239/s1. Figure S1: DU145 cells express a high level of embigin protein with high knockdown efficiency and transient or stable exogenous overexpression, Figure S2: Embigin promotes tumor growth in vivo, Figure S3: Embigin mediates prostate cancer cell survival under the condition of chemotherapy or oxidative stress independently of S100A4, Figure S4: Embigin mediates prostate cancer cell survival and maintains high cell migration under the condition of rapamycin treatment, Figure S5: S100A4/embigin mediates prostate cancer progression independently of AKT and MAPK signaling, Figure S6: Embigin does not alter the expression of ABCB1, ABCC1, and ABCC2 transporters and EMT-related genes but dephosphorylates mTOR at serine 2448, Figure S7: Embigin alters cancer-related genes that mostly associated with cell adhesion and extracellular matrix organization, Table S1: List of antibodies for WB and IHC analysis, Table S2: List of siRNA sequences targeting Embigin, Table S3: List of primers for qPCR analysis.

## Abbreviations

AMPK	Adenosine monophosphate-activated protein kinase
mTORC1	Mammalian target of rapamycin complex 1
NF-κB	Nuclear factor kappa-light-chain-enhancer of activated B cells
MMP9	Matrix Metalloproteinase 9
