# Supplementary material for: Embigin Promotes Prostate Cancer Progression by S100A4-Dependent and-Independent Mechanisms
Source: Cancers (Basel). 2018 Jul 23;10(7):239. doi: 10.3390/cancers10070239 (PMC6071117; doi:10.3390/cancers10070239)

Figure S1. DU145 cells express a high level of embigin protein with high knockdown efficiency and transient or stable exogenous overexpression.

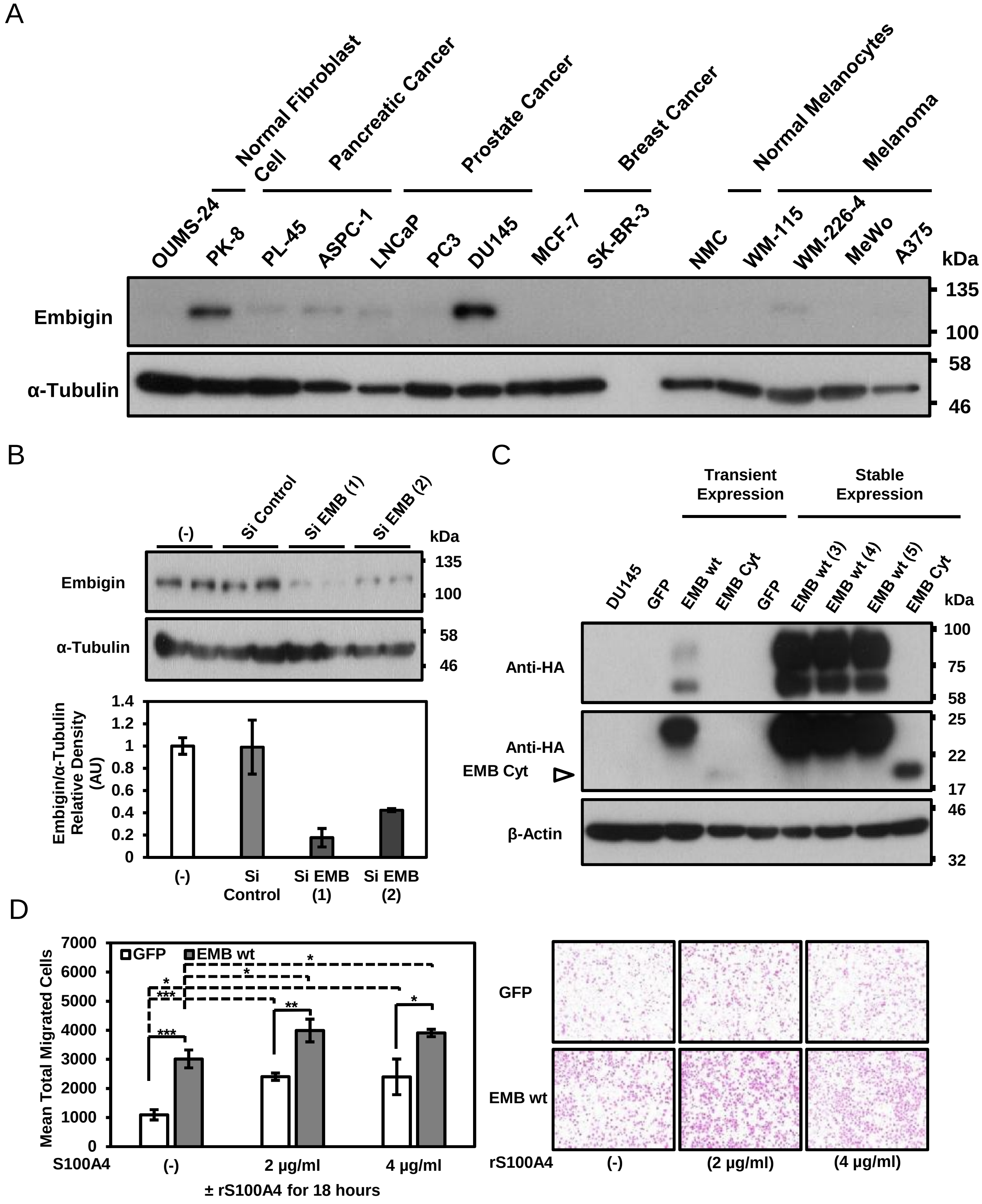

Figure S2. Embigin promotes tumor growth *in vivo*.

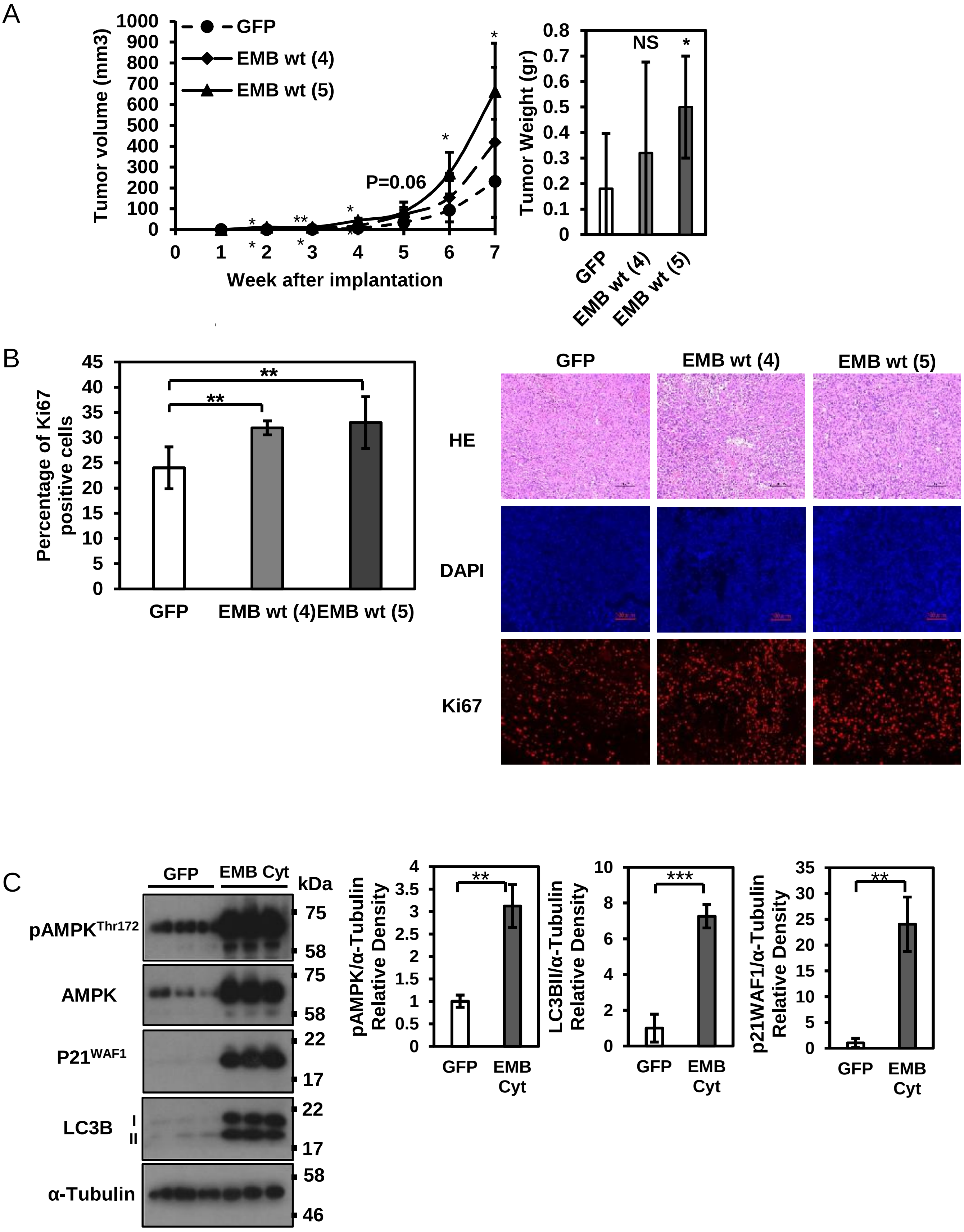

Figure S3. Embigin mediates prostate cancer cell survival under the condition of chemotherapy or oxidative stress independently of S100A4

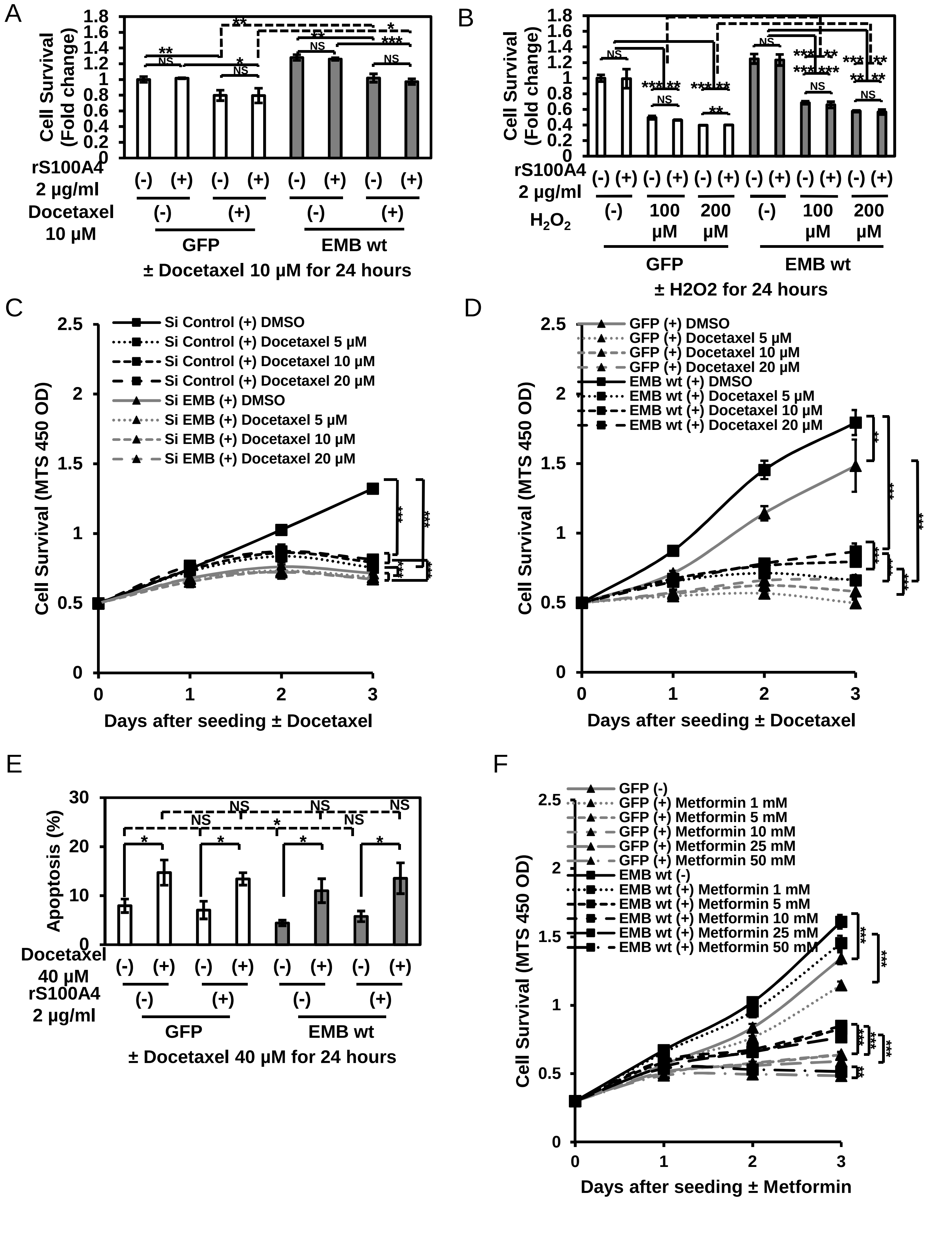

Figure S4. Embigin mediates prostate cancer cell survival
and maintains high cell migration under the condition of rapamycin treatment.

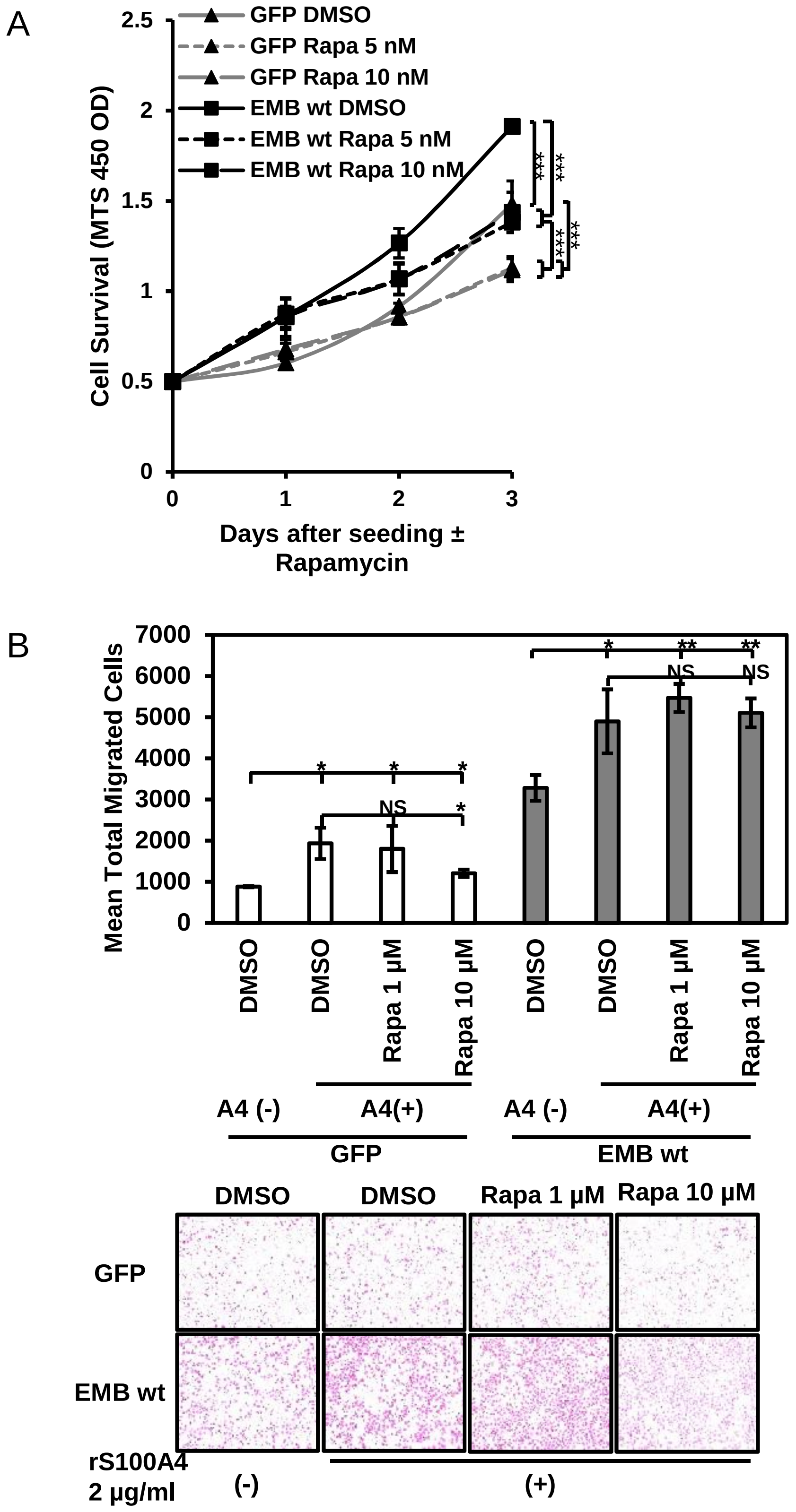

Figure S5. S100A4/embigin mediates prostate cancer progression Independently of AKT and MAPK signaling.

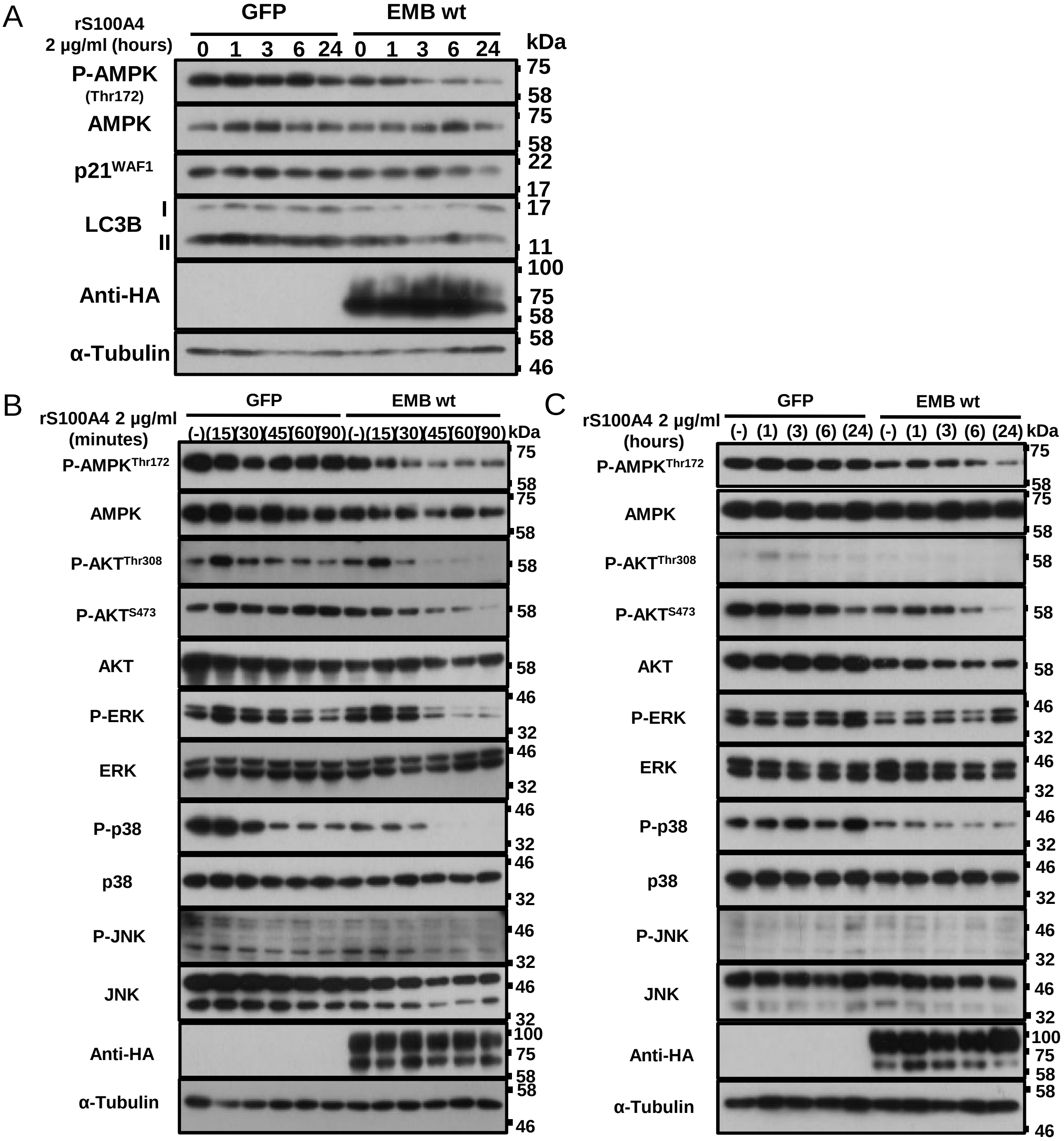

Figure S6. Embigin does not alter the expression of ABCB1, ABCC1, and ABCC2 transporters and EMT-related genes but dephosphorylates mTOR at Serine 2448.

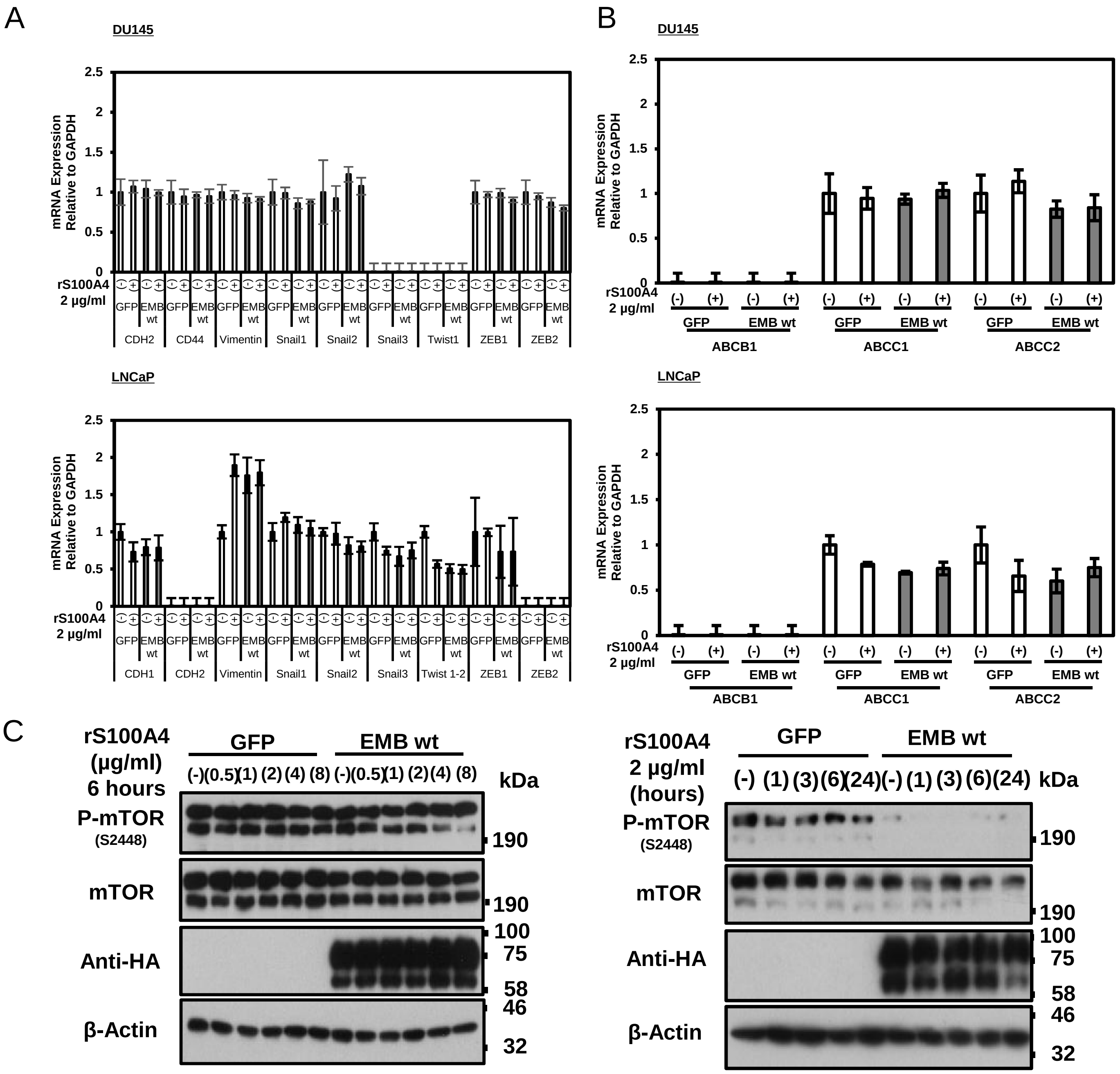

Figure S7. Embigin alters cancer-related genes that mostly associated with cell adhesion and extracellular matrix organization.

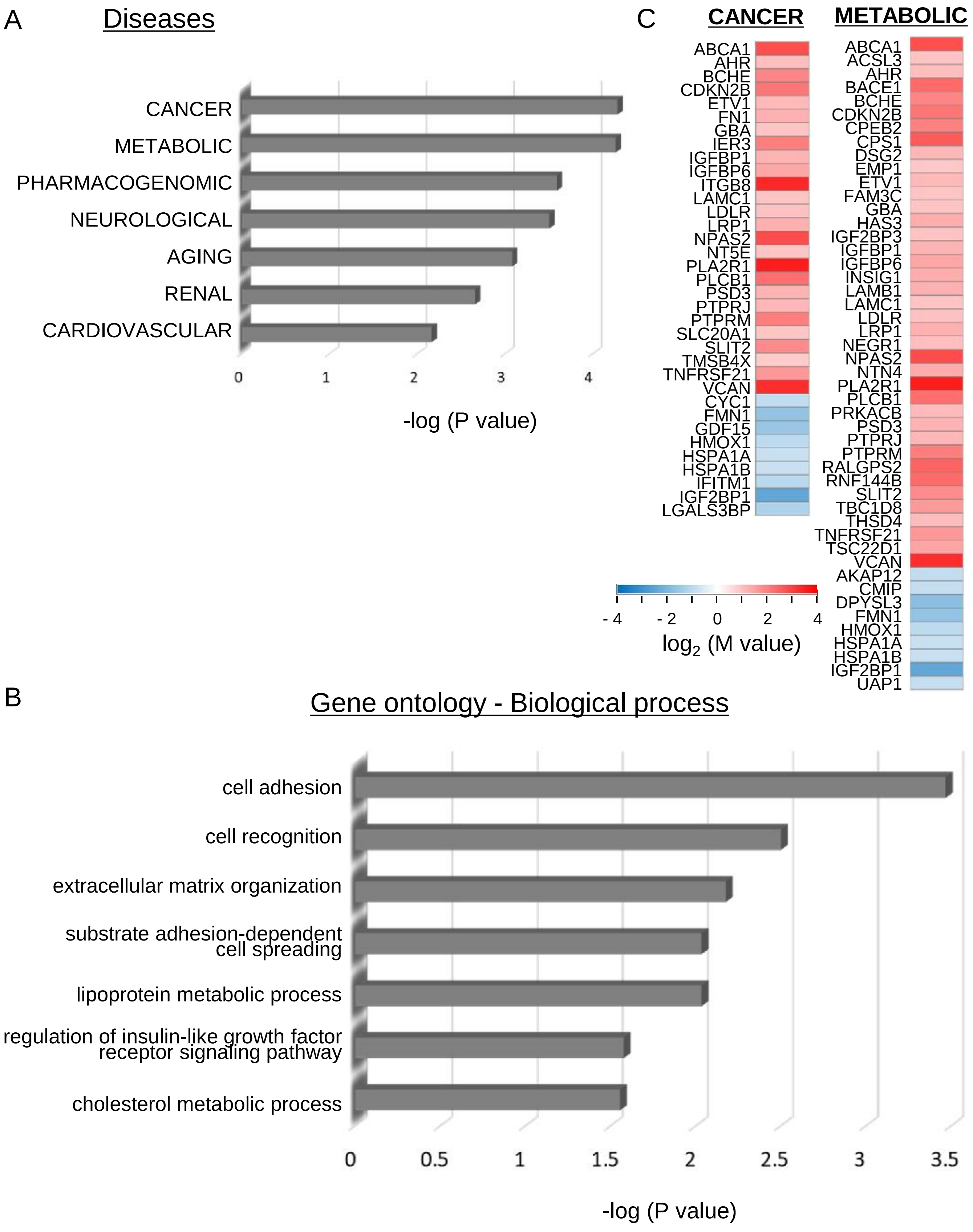

Supplement: Supplementary file 1 [file cancers-10-00239-s001.zip › Supplementary figure for Cancers.pdf]
