# Supplementary material for: Embigin Promotes Prostate Cancer Progression by S100A4-Dependent and-Independent Mechanisms
Source: Cancers (Basel). 2018 Jul 23;10(7):239. doi: 10.3390/cancers10070239 (PMC6071117; doi:10.3390/cancers10070239)
Supplement: Supplementary file 1 [file cancers-10-00239-s001.zip › Supporting information for Cancers.docx]

**Supplementary Figure Legends and Tables.**

1. **Supplementary Figure Legends**

**Figure S1. DU145 cells express a high level of embigin protein with high knockdown efficiency and transient or stable exogenous overexpression.** (A) Protein expression of embigin in pancreatic, prostate, and breast cancer and malignant melanoma cell lines determined by WB analysis. (B) Western blot analysis of DU145 cells that were transfected with siRNA control and two different siRNAs targeting *EMB*. (C) Western blot analysis of DU145 cells that were transiently or stably overexpressed with GFP, embigin wild-type (EMB wt), and embigin cytoplasmic tail (EMB Cyt). (D) Migration assay of DU145 stably overexpressing GFP and embigin wild-type that were stimulated to 2 and 4 µg/ml of rS100A4 for 18 hours (n=3). DU145 cells migration ability was upregulated by embigin in S100A4-dependent manner at 2 µg/ml of rS100A4 as the optimal concentration. One representative image is shown for each group. Data are presented as means ± SD. NS, not significant; ^*^, *P*<0.05; ^**^, *P*<0.01; ^***^, *P*<0.001, Student’s *t* test.

**Figure S2. Embigin promotes tumor growth *in vivo*.** (A) An *in vivo* subcutaneous growth model of tumors derived from DU145 cells stably overexpressing embigin compared to those of tumors derived from GFP-control cells (n=5 mice per group). (B) Percentages of Ki67-positive cells in mouse tumors implanted with DU145 cells stably overexpressing EMB wt clones 4 and 5 compared to those of tumors derived from GFP-control cells as shown by immunohistochemistry (n=5). (C) Western blot analysis of mouse tumors derived from DU145 cells stably overexpressing the embigin cytoplasmic tail (EMB Cyt) compared to those of tumors derived from GFP-control cells (n=3). Data are presented as means ± SD. NS, not significant; ^*^, *P*<0.05; ^**^, *P*<0.01; ^***^, *P*<0.001, Student’s *t* test.

**Figure S3**. **Embigin mediates prostate cancer cell survival under the condition of chemotherapy or oxidative stress independently of S100A4.** (A-B) LNCaP cells transiently overexpressing GFP and embigin wild-type (wt) that were stimulated with or not stimulated with 2 µg/ml of rS100A4 and 10 µM of docetaxel (A) or 100 or 200 µM of H_2_O_2_ (B) as evaluated by the MTS assay (n = 3). (C) MTS assay of DU145 cells with transient knockdown of embigin by siRNA compared with that of siRNA control-treated cells stimulated with 5, 10, and 20 µM of docetaxel (n = 6). (D) MTS assay of DU145 cells stably overexpressing embigin compared with that of GFP-control cells stimulated with 5, 10, and 20 µM of docetaxel (n = 6). (E) DU145 cells transiently overexpressing GFP and embigin wild-type (wt) that were stimulated with or not stimulated with 2 µg/ml of rS100A4 and high concentration of docetaxel (40 µM) as analyzed by Hoechst staining (n = 3). (F) MTS assay of DU145 cells stably overexpressing GFP and embigin wild-type (wt) that were treated with metformin (1-50 mM). Data are presented as means ± SD. NS, not significant; ^*^, *P*<0.05; ^**^, *P*<0.01; ^***^, *P*<0.001, Student’s *t* test.

**Figure S4. Embigin mediates prostate cancer cell survival and maintains high cell migration under the condition of rapamycin treatment.** (A) MTS assay of DU145 cells stably overexpressing GFP and embigin wild-type (wt) that were stimulated with 5 and 10 nM of rapamycin (n = 8). (B) Migration assay of DU145 cells stably overexpressing GFP and embigin wild-type that were treated with or not treated with 2 µg/ml of rS100A4 and 5-10 µM of rapamycin for 18 hours (n=3). One representative image is shown for each group. Data are presented as means ± SD. NS, not significant; ^*^, *P*<0.05; ^**^, *P*<0.01; ^***^, *P*<0.001, Student’s *t* test.

**Figure S5. S100A4/embigin mediates prostate cancer progression independently of AKT and MAPK signaling.** (A) Western blot analysis of LNCaP cells transiently overexpressing GFP and embigin wild-type (wt) that were stimulated with 2 µg/ml of rS100A4 for 1, 3, 6, and 24 hours. (B-C) Western blot analysis of DU145 cells stably overexpressing GFP and embigin wild-type (wt) stimulated with 2 µg/ml of rS100A4 for 15-90 minutes (B) or 1, 3, 6, and 24 hours (C).

**Figure S6. Embigin does not alter the expression of ABCB1, ABCC1, and ABCC2 transporters and EMT-related genes but dephosphorylates mTOR at serine 2448.** (A-B) Quantitative PCR analysis of DU145 or LNCaP cells transiently overexpressing GFP or embigin wild-type (wt) that were stimulated with or not stimulated with 2 µg/ml of rS100A4 for 24 hours showed no differences in the mRNA expression of EMT-related transcription factors (A) or ABCB1, ABCC1, and ABCC2-transporters genes relative to *Tbp* as an internal control (B). Data are presented as means ± SD. (C) Western blot analysis of DU145 cells stably overexpressing GFP and embigin wild-type (wt) stimulated with increasing concentrations of rS100A4 (0.5-8 µg/ml) for 6 hours (left panel) or with 2 µg/ml of rS100A4 for 1, 3, 6, and 24 hours (right panel).

**Figure S7. Embigin alters cancer-related genes that mostly associated with cell adhesion and extracellular matrix organization.** Total RNA was extracted from DU145 cells stably overexpressing embigin and fom GFP-control cells with ISOGEN (Nippon Gene, Tokyo, Japan) according to the manufacturer’s instructions. The isolated RNA was subjected to RNA-sequencing-based wide analysis of gene expression (Bioengineering Lab, Kanagawa, Japan). (A) Functional enrichment analysis (P<0.03) was then performed for protein-coding RNAs in GAD_DISEASE_CLASS with all of the upregulated and downregulated genes. (B) For the upregulated genes, functional enrichment analysis (P<0.03) was also performed in GO analysis. (C) Heat maps are displayed for the selected genes, which are upregulated and downregulated in cancer and metabolic category of the disease clustering as indicated in (A). These analyses were performed using the database for annotation, visualization and integrated discovery (DAVID) v6.8 (<http://david.ncifcr.gov/>).

1. **Supplementary Tables**

**Supplementary Table 1. List of antibodies for WB and IHC analysis.**

| **No** | **Antibody** | **Manufacturer** |
| --- | --- | --- |
| 1 | mouse anti-HA tag (clone 6E2) | Cell Signaling Technology, Beverly, MA |
| 2 | mouse anti-Myc tag (clone 9B11) | Cell Signaling Technology, Beverly, MA |
| 3 | rabbit anti-AMPK-α monoclonal Ab | Cell Signaling Technology, Beverly, MA |
| 4 | rabbit anti-Phospho AMPK-α (T172) monoclonal Ab | Cell Signaling Technology, Beverly, MA |
| 5 | rabbit anti-GSK3β monoclonal Ab | Cell Signaling Technology, Beverly, MA |
| 6 | rabbit anti-Phopho GSK3β (S9) | Cell Signaling Technology, Beverly, MA |
| 7 | rabbit anti-p70S6K monoclonal Ab | Cell Signaling Technology, Beverly, MA |
| 8 | rabbit anti-Phospho p70S6K (T389) monoclonal Ab | Cell Signaling Technology, Beverly, MA |
| 9 | rabbit anti-mTOR monoclonal Ab | Cell Signaling Technology, Beverly, MA |
| 10 | rabbit anti-Phospho mTOR (S2448) monoclonal Ab | Cell Signaling Technology, Beverly, MA |
| 11 | rabbit anti-p65 polyclonal Ab | Cell Signaling Technology, Beverly, MA |
| 12 | anti-Phospho p65 monoclonal Ab | Cell Signaling Technology, Beverly, MA |
| 13 | rabbit anti-LC3B monoclonal Ab | Cell Signaling Technology, Beverly, MA |
| 14 | rabbit anti-Embigin monoclonal Ab | Sigma-Aldrich, St. Louis, MO |
| 15 | rabbit anti-p21 monoclonal Ab | Abcam, Burlingame, CA |
| 16 | rabbit anti-MMP9 monoclonal Ab | Chemicon International, Temecula, CA |
| 17 | mouse anti-human tubulin Ab | Sigma-Aldrich, St. Louis, MO |
| 18 | mouse anti-human β-actin Ab | Sigma-Aldrich, St. Louis, MO |
| 19 | rabbit anti-Phospho AKT (Thr308) monoclonal Ab | Cell Signaling Technology, Beverly, MA |
| 20 | rabbit anti-Phospho AKT (S473) monoclonal Ab | Cell Signaling Technology, Beverly, MA |
| 21 | rabbit anti- AKT monoclonal Ab | Cell Signaling Technology, Beverly, MA |
| 22 | rabbit anti-Phospho ERK1/2 (T202/Y204) monoclonal Ab | Cell Signaling Technology, Beverly, MA |
| 23 | rabbit anti-ERK1/2 monoclonal Ab | Cell Signaling Technology, Beverly, MA |
| 24 | rabbit anti-Phospho p38 (T180/Y182) monoclonal Ab | Cell Signaling Technology, Beverly, MA |
| 25 | rabbit anti-p38 monoclonal Ab | Cell Signaling Technology, Beverly, MA |
| 26 | mouse anti-Phospho JNK (T183/Y185) monoclonal Ab | Cell Signaling Technology, Beverly, MA |
| 27 | rabbit anti-JNK monoclonal Ab | Cell Signaling Technology, Beverly, MA |
| 28 | rabbit anti-PARP monoclonal Ab | Cell Signaling Technology, Beverly, MA |
| 29 | rabbit anti-cleaved PARP monoclonal Ab | Cell Signaling Technology, Beverly, MA |
| 30 | rabbit anti-cleaved caspase 3 monoclonal Ab | Cell Signaling Technology, Beverly, MA |
| 31 | mouse anti-Ki67 monoclonal Ab | DAKO, Santa Clara, CA |

**Supplementary Table 2. List of siRNA sequences targeting *Embigin*.**

| **No** | **siRNA** | **Sense** | **Antisense** |
| --- | --- | --- | --- |
| 1 | siEMB (1) | GUGAACAACUUGAGAAUAAtt | UUAUUCUCAAGUUGUUCACca |
| 2 | siEMB (2) | GGAACAUAUGCUAACGAAAtt | UUUCGUUAGCAUAUGUUCCat |

**Supplementary Table 3. List of primers for qPCR analysis.**

| **No** | **Gene** | **Forward primer** | **Reverse primer** |
| --- | --- | --- | --- |
| 1 | ZEB1 | ggaggatgacacaggaaagg | tctgcatctgactcgcattc |
| 2 | ZEB2 | aggagctgtctcgccttg | ggcaaaagcatctggagttc |
| 3 | VIM (Vimentin) | gaccagctaaccaacgacaaa | gaagcatctcctcctgcaat |
| 4 | CDH1  (E-Cadherin) | cttactgcccccagaggat | gctggctcaagtcaaagtcc |
| 5 | CDH2  (N-Cadherin) | aatggatgaaagacccatccac | gagccactgccttcatagtcaa |
| 6 | SNAI1 (Snail1) | gctgcaggactctaatccaga | atctccggaggtgggatg |
| 7 | SNAI2 (Snail2) | acagcgaactggacacacat | gatggggctgtatgctcct |
| 8 | SNAI3 (Snail3) | cgctccttcctggtgaaa | agcaggcaccattgatttct |
| 9 | TWIST1 | ggcatcactatggactttctctatt | ggccagtttgatcccagtatt |
| 10 | CD44 | caacaacacaaatggctggt | ctgaggtgtctgtctctttcatct |
| 11 | EMB | aatagtggctgaggtgattctttt | gctcaaattctttcccctca |
| 12 | TBP (control) | gaacatcatggatcagaacaaca | atagggattccgggagtcat |
